# Chemotherapy-based versus chemotherapy-free stem cell mobilization (± plerixafor) in multiple myeloma patients: an Italian cost-effectiveness analysis

**DOI:** 10.1038/s41409-021-01251-8

**Published:** 2021-03-22

**Authors:** Carlo Lazzaro, Luca Castagna, Francesco Lanza, Daniele Laszlo, Giuseppe Milone, Luca Pierelli, Riccardo Saccardi

**Affiliations:** 1 Health Economist and Research Director, Studio di Economia Sanitaria, Milan, Italy; 2 Oncology and Haematology Unit, BMT section, Istituto Clinico Humanitas, Rozzano, Italy; 3 Hematology Section, Romagna Transplant Network, University Hospital “Santa Maria delle Croci”, Ravenna, Italy; 4 Stem Cell Mobilization and Collection Unit, IEO IRCCS, Milan, Italy; 5 Hematology and BMT Unit, Azienda Policlinico Vittorio Emanuele, Catania, Italy; 6 Department of Experimental Medicine, University “Sapienza”, Rome, Immune-hematology and Transfusion Medicine Unit, Azienda Ospedaliera San Camillo, Rome, Italy; 7 Department of Cellular Therapy and Transfusion Medicine, Careggi University Hospital, Florence, Italy

**Keywords:** Therapeutics, Stem-cell research

## Abstract

Given the availability and efficacy of the mobilizing agent plerixafor in augmenting hematopoietic progenitor cell mobilization with granulocyte colony-stimulating factor (G-CSF), there is a strong case for comparing the cost-effectiveness of mobilization with G-CSF + cyclophosphamide versus G-CSF alone. This study investigated the cost and effectiveness (i.e., successful 4 million-CD34^+^ collection) of G-CSF alone versus high-dose cyclophosphamide (4 g/m^2^) + G-CSF mobilization (± on-demand plerixafor) in patients with multiple myeloma (MM) eligible for autograft in Italy. A decision tree-supported cost-effectiveness analysis (CEA) model in MM patients was developed from the societal perspective. The CEA model compared G-CSF alone with cyclophosphamide 4 g/m^2^ + G-CSF (± on-demand plerixafor) and was populated with demographic, healthcare and non-healthcare resource utilization data collected from a questionnaire administered to six Italian oncohematologists. Costs were expressed in Euro (€) 2019. The CEA model showed that G-CSF alone was strongly dominant versus cyclophosphamide + G-CSF ( ± on-demand plerixafor), with incremental savings of €1198.59 and an incremental probability of a successful 4 million-CD34^+^ apheresis (+0.052). Sensitivity analyses confirmed the robustness of the base-case results. In conclusion, chemotherapy-free mobilization (± on-demand plerixafor) is a “good value for money” option for MM patients eligible for autograft.

## Introduction

In patients with multiple myeloma (MM) eligible for autologous stem cell (SC) transplant (SCT), SC are mobilized in peripheral blood for subsequent collection using granulocyte colony-stimulating factor (G-CSF) alone [1] or following chemotherapy with cyclophosphamide [1–4]. While various cyclophosphamide-based mobilization strategies are reported to mobilize SC while reducing toxicity [3] and improve SC yield, and prospective studies have used cyclophosphamide 1.0–7.0 g/m^2^ + G-CSF to mobilize SC in MM patients [2, 5–7], curative effects in MM have not been proven [8–10].

Plerixafor in combination with G-CSF has shown superiority in SC mobilization compared with G-CSF alone, mobilizing sufficient CD34^+^ cells to support SCT in MM patients [11–16]. Given the availability of plerixafor to augment SC mobilization with G-CSF, there is a strong case for comparing the cost-effectiveness of mobilization with G-CSF + high dose (4 g/m^2^) cyclophosphamide versus G-CSF alone [17–23]. This study compared the cost-effectiveness of chemotherapy-free and chemotherapy-based mobilization (± on-demand plerixafor) in MM patients eligible for autologous SCT at Italian oncohematology units.

## Methods

### Decision model

The opinions of a sample of convenience [24] of six Italian oncohematologists were elicited using a dedicated questionnaire to identify and quantify the parameters that were used for the cost-effectiveness analysis (CEA) model (Tables S1–S13) [25, 26].

Following the same approach as a previous CEA model of plerixafor in MM [23], the costs and probability of a successful 4 million-CD34^+^ collection in MM patients eligible for autologous SCT were calculated for SC mobilization with G-CSF alone versus cyclophosphamide 4 g/m^2^ + G-CSF ( ± on-demand plerixafor) using a decision tree model [27, 28]. As per the oncohematologists’ opinion, the time period for the decision tree ranged from SC mobilization to successful apheresis. Costs and outcomes of the subsequent autologous SCT were not included.

Since patients on cyclophosphamide show a higher risk of febrile neutropenia and/or need for red blood cell (RBC) and/or platelet (PLT) transfusions [27], the probability of these events, as well as achievement of successful apheresis, were estimated (Figures S1 and S2). The risk of death between mobilization and apheresis was not calculated.

### Probability of a successful CD34^+^ apheresis

The probability of successful apheresis (± on-demand plerixafor) was determined using both the oncohematologists’ opinion and the results of a previous Italian CEA of plerixafor in chemotherapy-based mobilization in MM [22]. The oncohematologists estimated the probability of administering on-demand plerixafor in chemotherapy-free mobilization was 47% to make it as effective as chemotherapy-based mobilization. Since the probability of a successful 4 million-CD34^+^ apheresis was 83.8% with on-demand plerixafor versus 70.2% without plerixafor in the previous CEA [22], the average probability of achieving the effectiveness outcome for chemotherapy-free mobilization (± on-demand plerixafor) was calculated as follows:

[(47% × 83.8%) + ([1 – 47%] × 70.2%)] = 76.5%

As the oncohematologists agreed that the probability of including plerixafor in cyclophosphamide + G-CSF mobilization was 9%, the average probability of a successful apheresis following chemotherapy-based mobilization (± on-demand plerixafor) was calculated as follows:

[(9% × 83.8%) + ([1 – 9%] × 70.2%)] = 71.4%

### Resource identification, quantification and valuation

As the analysis adopted the societal perspective [25, 26], healthcare and non-healthcare resources were valued. Healthcare resources funded by the Italian National Health Service (INHS) included: cyclophosphamide, G-CSF, and plerixafor and their administration (inpatient [cyclophosphamide], day-hospital [plerixafor], or at-home [G-CSF] settings); hospitalization for febrile neutropenia (with cyclophosphamide only) and RBC and/or PLT transfusions; central venous catheter (with cyclophosphamide only); apheresis (inpatient or outpatient setting); and SC handling (processing, cryopreservation, and thawing) (Tables S1–S7).

Healthcare services provided in the outpatient, day-hospital, or inpatient settings were costed according to INHS outpatient or hospital tariffs [29–31], assumed to be a reasonable approximation of the real costs borne by healthcare facilities (Tables S11–S13) [32]. As previously reported [33, 34], the total daily cost of hospitalization was calculated by dividing the current INHS tariff by the mean duration of inpatient stay (days) [30].

To avoid double-counting (i.e., costing the same healthcare resource twice [25]), whenever the healthcare procedure cost included in the INHS tariff (e.g., RBC transfusion) was calculated separately, the INHS tariff was halved to account for the hotel cost of inpatient stay or day-hospital only [25, 35]. Cyclophosphamide, plerixafor, and G-CSF were costed at their last available prices (Table S2) [36].

Opportunity costs (i.e., the economic value of the best alternative use of the same resources [32]) for missed apheresis due to poor mobilization were also estimated. Costs for patients and their families consisted of out-of-pocket expenses, patient’s productivity loss, and informal care provided by caregivers [25, 26]. Out-of-pocket expenses included all non-healthcare resources (e.g., transportation to and from healthcare facility and parking; Table S13) [37, 38].

One-half of patients were assumed to be employed (Table S4). The loss of working hours was valued using the gross wage rate (i.e., net wage + income taxes + social security contributions; Table S13) [39–41]. If the patient was unemployed or retired (i.e., ≥70 years old), working time was replaced by leisure time, which, like caregivers’ time, was costed at the take-home wage rate (net wage only; Table S13) [41, 42].

Costs were expressed in Euro (€) 2019 per patient and updated according to inflation rates for healthcare services or for general consumption whenever necessary [43]. As the time between mobilization and apheresis is expected not to exceed 1 year [5], costs and effectiveness were left undiscounted [17, 18, 25, 26, 44].

As this CEA model is not a clinical trial, study protocol approval by an ethics committee (including the questionnaire administered to oncohematologists) was not required by Italian legislation [45].

### Cost-effectiveness analysis

Differences in costs (incremental cost [ΔC]) and the probability of successful apheresis (incremental effectiveness [ΔE]) for the mobilization schemes were calculated and summarized using the incremental cost-effectiveness ratio (ICER) [25, 26] (Supplementary Materials Definition 1).

### Statistical analysis

The 95% confidence intervals (95% CIs) were calculated by assigning a statistical distribution to most of the parameters included in the CEA model (i.e., event probabilities, resource utilization, and unit costs) [27, 35, 46–48]. (Tables S3–S13). For parameters without a statistical distribution, a range was reported (Tables S1 and S2). No hypothesis testing was performed.

### Sensitivity analyses

The uncertainty of the base case ICER was addressed using one-way, probabilistic and scenario sensitivity analyses [25, 26, 49].

#### One-way sensitivity analysis

Parameters included in the one-way sensitivity analysis were changed one at time, holding the others at their base case values [25, 26, 49]. For each parameter, the baseline estimate was replaced with the lower and upper limits of the 95% CI or range [27, 35, 46, 47]. Results of one-way sensitivity analysis were reported on a tornado chart as horizontal bars that depart from the baseline ICER. The wider the horizontal bar, the higher the variation in the base case ICER due to parameter variation.

#### Probabilistic sensitivity analysis

Probabilistic sensitivity analysis assessed parameter uncertainty linked to the base case ICER via a 10,000-iteration Monte Carlo simulation [25–27, 46]. For each Monte Carlo iteration, a random value from the statistical distribution for each parameter was used to generate ΔC, ΔE, and the resulting ICER [27, 46].

The conjoint density of 10,000 pairs of ΔC and ΔE for the healthcare program that ranked first in the base case CEA (treatment) was plotted on the cost-effectiveness plane, a two-dimensional surface divided into four sectors [50]. Each sector implies different ICER interpretations: the North East (NE) sector includes the iterations for which treatment is more costly and more effective than the comparator; the North West (NW) sector shows those iterations for which treatment is more costly and less effective than (i.e., is strongly dominated by) the comparator; in the South West (SW) sector treatment is less costly and less effective than the comparator; and in the South East (SE) sector the treatment is less costly and more effective than (i.e., strongly dominates) the comparator. The share of Monte Carlo iterations that fall below and to the right of the threshold value is defined as cost-effective.

An algebraic manipulation of the ICER (Net Monetary Benefit; Supplementary Materials Definition 2) [27, 46] used the Monte Carlo simulation results to construct the cost-effectiveness acceptability curve (Supplementary Materials Definition 3) and cost-effectiveness acceptability frontier (Supplementary Materials Definition 4), which summarize the probability for each mobilization strategy to be cost-effective or optimal versus its comparator for a given set of threshold values [27, 46, 51, 52]. As drug costs were kept constant, they were not included in sensitivity analyses [46].

#### Scenario sensitivity analysis

Scenario sensitivity analyses was performed to determine the impact of variation of one or more parameters at time on the baseline ICER [25, 49].

## Results

### Decision tree

Regardless of the SC mobilization scheme used, patients were assumed to enter the CEA model at a mean age of 57.62 years (Table 1).

**Table 1:** Summary of non-monetary results of the cost-effectiveness analysis model.

Parameters	G-CSF (± on-demand PLX)	CTX + G-CSF (± on-demand PLX)
Demographic and anthropometric parameters		
Age, years, mean (range)	57.62 (51.38, 62.38)	57.62 (51.38, 62.38)
Bodyweight, kg, mean (95% CI)	70.00 (26.34, 134.61)^a^	70.00 (26.34, 134.61)^a^
Height, cm, mean (95% CI)	170.00 (136.68, 203.32)^a^	170.00 (136.68, 203.32)^a^
Mobilization parameters, mean (95% CI)		
Number of vials of on-demand PLX	0.71 (0.00, 3.04)^a^	0.14 (0.00, 1.22)^a^
Days of hospitalization for CTX administration	0	2.00 (0.75, 3.85)^a^
Days of hospitalization for febrile neutropenia	0	0.35 (0.00, 2.04)^a^
Number of RBC transfusions	0	0.24 (0.00, 1.68)^a^
Number of PLT transfusions	0	0.12 (0.00, 1.10)^a^
Apheresis parameters, mean (95% CI)		
Performed apheresis sessions	2.00 (0.75, 3.85)^a^	1.60 (0.60, 3.08)^a^
Missed apheresis sessions due to poor mobilization	0.48 (0.18, 0.92)^a^	0.95 (0.36, 1.83)^a^
Employment (patients only)^b^		
Productivity losses, hours, mean (95% CI)	11.47 (0.01, 58.96)^a^	78.08 (17.50, 182.84)^a^
Informal care		
Informal care, hours, mean (95% CI)	11.34 (0.01, 58.29)^a^	76.46 (16.85, 180.59)^a^

*CI* confidence interval, *CTX* cyclophosphamide, *G-CSF* granulocyte colony-stimulating factor, *PLT* platelet, *PLX* plerixafor, *RBC* red blood cell.

^a^Gamma distribution 95% CI [27, 47].

^b^If the patient was unemployed, not engaged in housekeeping or retired (≥70 years old), working time was replaced by leisure time.

In this model, patients undergoing chemotherapy-free and chemotherapy-based mobilization received a mean of 0.71 and 0.14 vials of on-demand plerixafor, respectively (Table 1). No other healthcare resource was used by patients who did not receive cyclophosphamide. Use of healthcare resources for those mobilized with cyclophosphamide + G-CSF ( ± on-demand plerixafor) included time for inpatient cyclophosphamide administration, increased inpatient stay for febrile neutropenia, and administration of RBC and PLT transfusions (Table 1).

Patients on chemotherapy-free and chemotherapy-based mobilization underwent 2.00 and 1.60 apheresis sessions, respectively, but missed 0.48 and 0.95 apheresis sessions due to poor mobilization. On average, patients on chemotherapy-free and chemotherapy-based mobilization lost 11.47 and 78.08 working hours, respectively, to receive healthcare services and needed 11.34 and 76.46 hours of informal care.

### Cost and cost-effectiveness analysis

The total societal cost for G-CSF alone and cyclophosphamide + G-CSF ( ± on-demand plerixafor) was €8039.85 and €9238.44 per patient, respectively (Table 2). The INHS-funded per patient cost for G-CSF alone and cyclophosphamide + G-CSF ( ± on-demand plerixafor) reached €7494.27 and €5984.30, respectively (93.21% and 64.77% of the overall cost). The out-of-pocket expenses were €122.29 and €321.90 per patient, respectively (1.52% versus 3.49% of overall cost), and productivity losses plus informal care costs were €423.30 and €2932.24, respectively (5.27% versus 31.74% of overall cost). For both healthcare programs, the cost-drivers were drugs prescribed during mobilization (61.29% and 20.86% of overall cost).

**Table 2:** Base case analysis – Costs per patient (€2019).

Cost items	G-CSF (± on-demand PLX)	Proportion of overall cost (%)^a^	CTX + G-CSF (± on-demand PLX)	Proportion of overall cost (%)^a^
INHS cost				
Mobilization				
Drugs	€4927.05	61.29	€1926.87	20.86
* CTX*	€*0.00*	*–*	€*128.13*	*6.65*
* G-CSF*	€*1332.52*	*27.04*	€*1110.43*	*57.63*
* PLX*	€*3594.53*	*72.96*	€*688.31*	*35.72*
Administration	€92.02	1.14	€439.69	4.76
* CTX*	€*0.00*	*–*	€*422.07*	*96.00*
* G-CSF*	€*0.00*	*–*	€*0.00*	*0.00*
* PLX*	€*92.02*	*100*	€*17.62*	*4.00*
Full blood count	€4.76	0.06	€4.76	0.05
Central venous catheter	€0.00	–	€150.58	1.63
Febrile neutropenia	€0.00	–	€149.15	1.61
Transfusions	€0.00	–	€739.27	8.00
* RBC*	€*0.00*	*–*	€*495.89*	*67.08*
* PLT*	€*0.00*	*–*	€*243.38*	*32.92*
Apheresis				
Procedures	€1144.45	14.23	€1247.99	13.51
* Flow cytometry*	€*51.00*	*4.46*	€*51.00*	*4.09*
* Full blood count*	€*4.76*	*0.42*	€*12.02*	*0.96*
* Apheresis*	€*877.98*	*76.72*	€*767.92*	*61.53*
* Missed apheresis*	€*210.72*	*18.41*	€*417.04*	*33.42*
Stem cell handling	€1326.00	16.49	€1326.00	14.35
* Processing*	€*668.00*	*50.38*	€*668.00*	*50.38*
* Freezing*	€*510.00*	*38.46*	€*510.00*	*38.46*
* Thawing*	€*148.00*	*11.16*	€*148.00*	*11.16*
Total (A)	€7494.27	93.21%	€5984.3	64.77%
Patient and their family cost (out-of-pocket expenses)				
Transportation	€92.29	1.15	€242.7	2.63
Parking	€30.00	0.37	€79.2	0.86
Total (B)	€122.29	1.52%	€321.9	3.49%
Patient and their family cost (Patient and care-giver’s time)				
Patient loss of working days^b^	€249.17	3.10	€1695.84	18.36
Informal care	€174.13	2.17	€1236.4	13.38
Total (C)	€423.3	5.27%	€2932.24	31.74%
Overall cost (A+B+C)	€8039.85	100.00%	€9238.44	100.00%

*CTX* cyclophosphamide, *G-CSF* granulocyte colony-stimulating factor, *INHS* Italian National Health Service, *PLT* platelets, *PLX* plerixafor, *RBC* red blood cells.

^a^The percentage of each category/item cost on the overall cost is reported in normal font. Italic font denotes the percentage of each sub-item on the category/item cost.

^b^If the patient was unemployed, not engaged in housekeeping or retired (i.e., ≥70 years old), working time was replaced by leisure time.

In the CEA, G-CSF alone versus cyclophosphamide + G-CSF ( ± on-demand plerixafor) was associated with a ΔC of − €1198.59 (i.e., incremental saving) and an ΔE of +0.052 (i.e., an incremental probability of a successful 4 million-CD34^+^ apheresis), indicating that G-CSF alone was strongly dominant over cyclophosphamide + G-CSF ( ± on-demand plerixafor; Table 3).

**Table 3:** Base case cost-effectiveness analysis (€2019).

Mobilization schemes	Cost	Effectiveness^a^	ΔC	ΔE	ICER (ΔC/ΔE)
CTX 4 g/m^2^ + G-CSF ± on-demand PLX	€ 9238.44	0.714	–	–	–
G-CSF ± on-demand PLX	€ 8039.85	0.766	−€ 1198.59	0.052	strongly dominant

*ΔC* incremental cost, *ΔE* incremental effectiveness, *CTX* cyclophosphamide, *G-CSF* granulocyte colony-stimulating factor, *ICER* incremental cost-effectiveness ratio for G-CSF ± on-demand PLX vs CTX 4 g/m^2^ + G-CSF ± on-demand PLX, *PLX* plerixafor.

^a^Probability of a successful 4 million-CD34^+^ apheresis.

### Sensitivity analyses

#### One-way sensitivity analysis

**Fig. 1:**
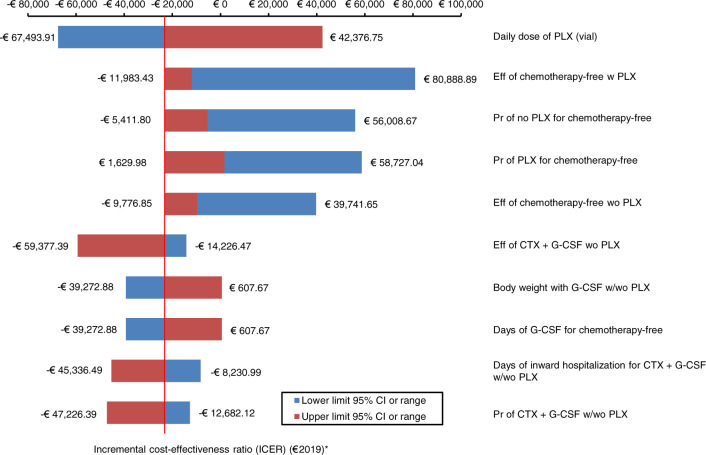
Tornado chart showing one-way sensitivity analysis (€2019) of G-CSF ± on-demand plerixafor versus cyclophosphamide 4 g/m^2^ + G-CSF ± on-demand plerixafor. The base case ICER for G-CSF ± on-demand plerixafor was –€23,192.47 (indicated by the red vertical line) and was strongly dominant. *CTX* cyclophosphamide, *Eff* effectiveness, *G-CSF* granulocyte colony-stimulating factor, *ICER* incremental cost-effectiveness ratio, *PLX* plerixafor, *Pr* probability, *w* with, *wo* without.

As shown in the tornado chart (Fig. 1), when expressed in percentages, the largest variations in baseline ICER were observed with changes in the number of plerixafor vials administered (from –282.72% to +191.02% versus base case ICER). Variation in the probability of plerixafor administration affected baseline findings moderately (from −45.32% to +103.63% versus base case ICER).

#### Probabilistic sensitivity analysis

**Fig. 2:**
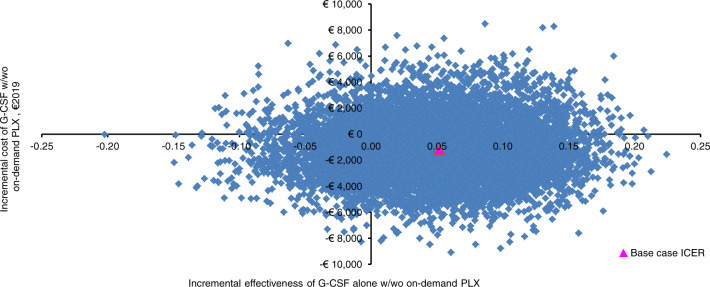
Cost-effectiveness plane showing probabilistic sensitivity analysis (€2019) of G-CSF ± on-demand plerixafor versus cyclophosphamide 4 g/m^2^ + G-CSF ± on-demand plerixafor. The base case ICER for G-CSF ± on-demand plerixafor was strongly dominant. G-CSF granulocyte colony-stimulating factor, ICER incremental cost-effectiveness ratio, PLX plerixafor, w with, wo without.

Of the 10,000 Monte Carlo iterations, chemotherapy-free mobilization was more costly and more effective than chemotherapy-based mobilization (NE sector of the cost-effectiveness plane) in 2314 (23.14%). For 440 iterations (4.40%), G-CSF alone (± on-demand plerixafor) was more costly and less effective than chemotherapy-based mobilization (NW sector). In 5992 iterations (59.92%), chemotherapy-free mobilization was less costly and more effective (SE sector) and strongly dominated chemotherapy-based mobilization. Eventually, for 1254 iterations (12.54%), chemotherapy-free mobilization was less costly and less effective (SW sector) than chemotherapy-based mobilization (Fig. 2).

**Fig. 3:**
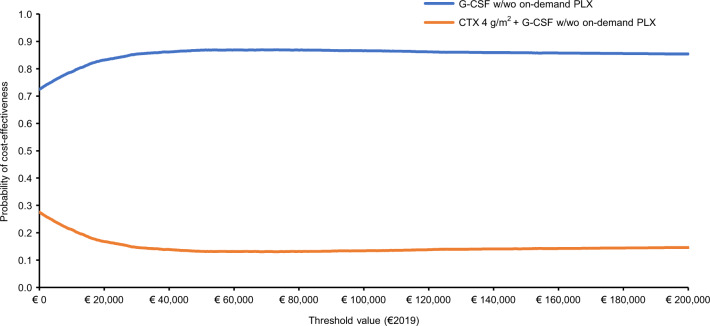
Cost-effectiveness acceptability curve showing probabilistic sensitivity analysis (€2019) of G-CSF ± on demand plerixafor versus cyclophosphamide 4 g/m^2^ + G-CSF ± on-demand plerixafor. The base case ICER for G-CSF ± on-demand plerixafor was strongly dominant. CTX cyclophosphamide, G-CSF granulocyte colony-stimulating factor, PLX plerixafor, w with, wo without.

The likelihood of G-CSF (± on-demand plerixafor) being cost-effective was 72.46%, 84.24%, and 86.12% when the threshold was €0, €25,000, and €40,000, respectively (Fig. 3).

**Fig. 4:**
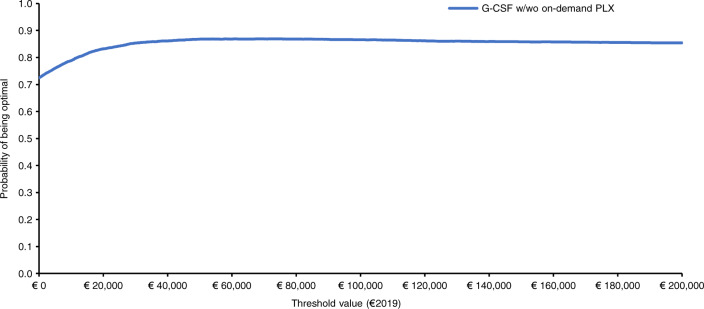
Cost-effectiveness acceptability frontier showing probabilistic sensitivity analysis (€2019) for GCS-F ± on-demand plerixafor versus cyclophosphamide 4 g/m^2^ + G-CSF ± on-demand plerixafor. The base case ICER for GCS-F ± on-demand plerixafor was strongly dominant. GCS-F ± plerixafor was the optimal healthcare program from a threshold value of €0.00 onwards. G-CSF granulocyte colony-stimulating factor, PLX plerixafor, w with, wo without.

The cost-effectiveness acceptability frontier showed that G-CSF ( ± on-demand plerixafor) starts to be the optimal alternative from a societal willingness to pay (WTP) of €0 onwards (Fig. 4).

#### Scenario sensitivity analysis

For most of the assumptions tested in the scenario sensitivity analysis, the chemotherapy-free mobilization regimen retained its strong dominance (Table 4). When both mobilization regimens were assumed to be equally effective, the chemotherapy-free regimen was weakly dominant (i.e., less costly but equally effective) versus the chemotherapy-based regimen.

**Table 4:** Scenario sensitivity analysis (€2019).

Mobilization schemes	Cost	Effectiveness^a^	ΔC	ΔE	ICER (ΔC/ΔE)
Base case analysis					
CTX 4 g/m^2^ + G-CSF ± on-demand PLX	€ 9238.44	0.714	–	–	–
G-CSF ± on-demand PLX	€ 8039.85	0.766	−€ 1198.59	0.052	strongly dominant
Scenario sensitivity analysis – Same effectiveness for chemo-free and chemo-based mobilization regimens					
CTX 4 g/m^2^ + G-CSF ± on-demand PLX	€ 9238.44	0.766	–	–	–
G-CSF ± on-demand PLX	€ 8039.85	0.766	−€ 1198.59	0.000	weakly dominant
Scenario sensitivity analysis – Reversed effectiveness for chemo-free and chemo-based mobilization regimens					
CTX 4 g/m^2^ + G-CSF ± on-demand PLX	€ 9238.44	0.766	–	–	–
G-CSF ± on-demand PLX	€ 8039.85	0.714	−€ 1198.59	- 0.052	€ 23,049.81
Scenario sensitivity analysis – CTX administration costed with DH tariff for all patients on chemo-based mobilization regimen					
CTX 4 g/m^2^ + G-CSF ± on-demand PLX	€ 8438.32	0.714	–	–	–
G-CSF ± on-demand PLX	€ 8708.42	0.766	€ 270,09	0.052	€ 5226.29
Scenario sensitivity analysis – Reversed percentages for inward and outpatient management of neutropenia for all patients on chemo-based mobilization regimen					
CTX 4 g/m^2^ + G-CSF ± on-demand PLX	€ 9114.88	0.714			
G-CSF ± on-demand PLX	€ 8039.85	0.766	−€ 1075.03	0.052	strongly dominant
Scenario sensitivity analysis – Central venous catheter for all patients on chemo-free mobilization regimen undegoing apheresis					
CTX 4 g/m^2^ + G-CSF ± on-demand PLX	€ 9238.44	0.714	–	–	–
G-CSF ± on-demand PLX	€ 8110.62	0.766	−€ 1127.81	0.052	strongly dominant
Scenario sensitivity analysis – Outpatient apheresis for all patients on chemo-based mobilization regimen					
CTX 4 g/m^2^ + G-CSF ± on-demand PLX	€ 9142.26	0.714	–	–	–
G-CSF ± on-demand PLX	€ 8039.85	0.766	−€ 1102.41	0.052	strongly dominant

*ΔC* incremental cost, *ΔE* incremental effectiveness, *CTX* cyclophosphamide, DH day-hospital, *G-CSF* granulocyte colony-stimulating factor, *ICER* incremental cost-effectiveness ratio for G-CSF ± on-demand PLX vs CTX 4 g/m^2^ + G-CSF ± on-demand PLX, *PLX* plerixafor.

^a^Probability of a successful 4 million-CD34^+^ apheresis.

## Discussion

To our knowledge, this is the first Italian CEA to compare chemotherapy-free with chemotherapy-based mobilization (± on-demand plerixafor) in patients with MM using a decision model populated with parameters elicited from a panel of experienced oncohematologists. While our base-case CEA shows that G-CSF ( ± on-demand plerixafor) is strongly dominant, probabilistic sensitivity analysis suggests that this mobilization regimen has a high probability of being cost-effective.

Previous Italian studies have estimated healthcare resource utilization and costs of mobilization and collection of SC in MM, lymphoma, and other blood malignancies [53–55], and provided ICER for a successful apheresis in MM patients on chemotherapy-based mobilization [22]. However, these studies followed the hospital viewpoint (i.e. costs calculated only for the healthcare resources consumed by MM patients in the inpatient and day-hospital settings), whereas our CEA model adopted the wider societal perspective, as chemotherapy-based mobilization may affect patients’ (and their families’) resources and time more severely than chemotherapy-free schemes [18, 56].

Most of the economic evaluation of healthcare programmes comparing mobilization with versus without plerixafor focused on cost per quality-adjusted life year (QALY) gained. QALY is a disease non-specific, non-clinical effectiveness measure that weights patients’ survival for health-related quality of life (utility) [25, 26].

Cost-utility analyses performed on patients with non-Hodgkin’s lymphoma [57] or MM [58] reported incremental cost-utility ratios (ICUR) of US$14,735 and US$52,813, respectively, with plerixafor administration (2010 values). When assessing the sustainability of the cost per QALY gained, decision-makers should consider country-specific threshold values. WTPs of US$50,000 and US$100,000 per QALY gained are often quoted for North America [59, 60], whereas the National Institute for Health and Care Excellence (NICE) more recently confirmed an acceptability range of UK£20,000–UK£30,000 per QALY gained in England and Wales [61]. In 2009, the Italian Association of Health Economics proposed a WTP threshold similar to that set by NICE (€25,000–€40,000) [44]. However, the implicit threshold value adopted by the Italian Medicines Agency for reimbursing oncology drugs has been estimated at €87,330 (2014 values) [62].

Even though the cost per QALY gained remains the determinant for rationing in the healthcare sector [25, 26], our analysis may contribute to defining the economic sustainability of successful apheresis with chemotherapy-free mobilization (± on-demand plerixafor) in MM patients. This topic warrants further investigation, due to the lack of cost-effectiveness thresholds for clinical effectiveness outcomes (e.g., successful apheresis). The absence of a specific WTP for successful mobilization may be the main reason why one UK CEA on plerixafor concluded that no opinion on its affordability could be made (ICERs of UK£12,608–UK£15,450; year of currency not reported) [20]. Similarly, a Czech decision tree-supported CEA model reported a ΔE of 10% with G-CSF ± plerixafor versus G-CSF ( ± chemotherapy), with an ICER of US$11,803 for successful mobilization (2013 values) [23]. However, no recommendation was provided to the national decision-makers on whether or not plerixafor was a “good value for money” option [23]. In contrast, a model-based CEA highlighted the unaffordability for the Jordan healthcare system of G-CSF + plerixafor versus G-CSF ( ± cyclophosphamide) with an ICER of US$244,714 per successful apheresis (year of currency not reported) [17].

A US study comparing low-dose cyclophosphamide (1.5–2.0 g/kg/day) + G-CSF versus G-CSF + plerixafor in MM patients undergoing mobilization reported no significant difference in effectiveness with regard to collection of ≥2, ≥5, or ≥10 million CD34^+^ cells/kg [18]. In the same study, G-CSF + plerixafor had a ΔC of +US$6475.20 versus G-CSF + cyclophosphamide (total costs US$28,980.00 versus US$22,504.80; *p* = 0.001; 2012 values). As the study was conducted from the Medicare and Medicaid perspective, the cost difference was exclusively due to higher healthcare resource utilization with chemotherapy-free mobilization [18]. Further investigation from a societal perspective may reveal whether the increased costs for chemotherapy-based mobilization due to higher out-of-pocket expenses, patient’s time consumption, and informal care associated with cyclophosphamide administration bridge the economic gap between the two mobilization strategies [18]. In another US study in MM patients, mobilization with G-CSF + plerixafor was more costly (total cost US$28,980.00 versus US$19,626.50; *p* = 0.001; 2012 values) and more effective (higher percentage of 2–10 million CD34^+^ cells collected; *p* = 0.01–0.001) than G-CSF + low-dose cyclophosphamide [19]. Following the French healthcare system viewpoint, G-CSF + plerixafor was more costly (mean cost €10,958.00 versus €5097.00; *p* < 0.0001; 2012 values) and more effective (autologous SCT 86% versus 67%; *p* = 0.02) than high-dose cyclophosphamide in MM patients [63]. However, a CEA was not conducted in these studies.

Furthermore, with the currently available therapeutic options for MM patients, including agents that prevent exposure to conventional chemotherapy and their associated systemic toxicity, the placement and role of cyclophosphamide as a “pure” SC mobilizing agent appears questionable.

Our research has three main limitations. First, the parameters of the CEA model were obtained from a panel of Italian oncohematologists who were introduced to each other during a meeting to discuss the feasibility of this study. Therefore, the Delphi method [64] was not applied, as the requirement of panelist anonymity could not be met. Second, following previously research [23, 58], we performed a model-based CEA rather than an empirical one using clinical trial data [20–22]. Given the advantages and disadvantages of model-based versus empirical studies [25–27, 65, 66], the robustness of our base-case findings was checked and confirmed via three sensitivity analyses. Future empirical CEAs on chemotherapy-based versus chemotherapy-free mobilization (± on-demand plerixafor) in MM patients will hopefully provide more reliable ICERs [56]. Third, the mean age of the notional patients entering the Markov model is lower than the one reported in other research [3]. However, one-way sensitivity analysis proved that patients’ age was not among the ten parameters that caused the most relevant departures from the base-case ICER.

In conclusion, our study shows that, from a societal perspective, chemotherapy-free mobilization (± on-demand plerixafor) is a cost-effective (and possibly strongly dominant) healthcare program for patients with MM who are eligible for autologous SCT in Italy.

## Supplementary information

Supplementary materials
