# Supplementary material for: Chemotherapy-based versus chemotherapy-free stem cell mobilization (± plerixafor) in multiple myeloma patients: an Italian cost-effectiveness analysis
Source: Bone Marrow Transplant. 2021 Mar 22;56(8):1876–87. doi: 10.1038/s41409-021-01251-8 (PMC8338551; doi:10.1038/s41409-021-01251-8)
Supplement: Supplementary file 1 — Supplementary materials [file 41409_2021_1251_MOESM1_ESM.docx]

**Chemotherapy-based versus chemotherapy-free stem cell mobilization (± plerixafor) in multiple myeloma patients: an Italian cost-effectiveness analysis**

**Authors:** Carlo Lazzaro^1^, Luca Castagna^2^, Francesco Lanza^3^, Daniele Laszlo^4^, Giuseppe Milone^5^, Luca Pierelli^6^, Riccardo Saccardi^7^

**Affiliations:** ^1^Health economist and research director, Studio di Economia Sanitaria, Milan, Italy; ^2^Oncology and Haematology Unit, BMT section, Istituto Clinico Humanitas, Rozzano, Italy; ^3^Hematology Section, Romagna Transplant Network, University Hospital “Santa Maria delle Croci”, Ravenna, Italy; ^4^Stem cell mobilization and collection Unit, IEO IRCCS, Milan, Italy; ^5^Hematology and BMT Unit, Azienda Policlinico Vittorio Emanuele, Catania, Italy; ^6^Department of Experimental Medicine, University “Sapienza”, Rome, Immune-hematology and Transfusion Medicine Unit, Azienda Ospedaliera San Camillo, Rome, Italy; ^7^Department of Cellular Therapy and Transfusion Medicine, Careggi University Hospital, Florence, Italy.

# Supplementary Material

## Section A: G-CSF alone vs cyclophosphamide 4 g/m^2^ + G-CSF (± on demand plerixafor)

*G-CSF* granulocyte colony-stimulating factor.

## Supplementary Table S1 Parameters with no statistical distribution for base case and sensitivity analyses (one-way and probabilistic)

| **Parameter number** | **Parameter**  **name** | **Spreadsheet**  **(point estimate cell)** | **Parameter**  **description** | **Point estimate** | **Range** | | **Source** |
| --- | --- | --- | --- | --- | --- | --- | --- |
|  |  |  |  |  | **LL** | **UL** |  |
| **G-CSF alone vs CTX 4 g/m^2^ + G-CSF (± on-demand PLX)** | | | | | | | |
| **Demographics** | | | | | | | |
| 1 | Age | Parameters (L19) | Patients’ age when entering the model | 57.62 | 51.38 | 62.38 | ^1^ |
| **Healthcare resources: Probability of utilization and related provision setting for mobilization** | | | | | | | |
| 2 | Pr_CTX_for_CTX_plus_G_CSF_w_wo_PLX_MOB | Parameters (L22) | Probability of CTX for patients on CTX 4 g/m^2^ + G-CSF ± on-demand PLX | 1.000 | 1.000 | 1.000 | Oncohematologists’ opinion |
| 3 | Pr_GCSF_for_CTX_plus_G_CSF_w_wo_PLX_MOB | Parameters (L27) | Probability of G-CSF for patients on CTX 4 g/m^2^ + G-CSF ± on-demand PLX | 1.000 | 1.000 | 1.000 | Oncohematologists’ opinion |
| 4 | Pr_Inw_Hosp_CTX_CTX_plus_G_CSF_w_wo_PLX_MOB | Parameters (L28) | Probability of inward hospitalization for CTX administration for patients on CTX 4 g/m^2^ + G-CSF ± on-demand PLX | 1.000 | 1.000 | 1.000 | Oncohematologists’ opinion |
| 5 | Pr_DH_PLX_CTX_plus_G_CSF_w_wo_PLX_MOB | Parameters (L29) | Probability of DH access for PLX administration for patients on CTX 4 g/m^2^ + G-CSF ± on-demand PLX | 1.000 | 1.000 | 1.000 | Oncohematologists’ opinion |
| 6 | Pr_Full_blood_count_for_CTX_plus_GCSF_w_wo_PLX_MOB | Parameters (L30) | Probability of full blood count for patients on CTX 4 g/m^2^ + G-CSF ± on-demand PLX | 1.000 | 1.000 | 1.000 | Oncohematologists’ opinion |
| 7 | Pr_CVC_for_CTX_plus_G_CSF_w_wo_PLX_MOB | Parameters (L31) | Probability of CVC for patients on CTX 4 g/m^2^ + G-CSF ± on-demand PLX | 1.000 | 1.000 | 1.000 | Oncohematologists’ opinion |
| 8 | Pr_GCSF_for_Chemo_free_MOB | Parameters (L37) | Probability of G-CSF for patients on G-CSF ± PLX on demand | 1.000 | 1.000 | 1.000 | Oncohematologists’ opinion |
| 9 | Pr_DH_PLX_Chemo_free_MOB | Parameters (L38) | Probability of DH access for PLX administration for patients on G-CSF ± on-demand PLX | 1.000 | 1.000 | 1.000 | Oncohematologists’ opinion |
| 10 | Pr_Full_blood_count_for_Chemo_free_MOB | Parameters (L39) | Probability of full blood count for patients on G-CSF ± on-demand PLX | 1.000 | 1.000 | 1.000 | Oncohematologists’ opinion |
| **Healthcare resources: probability of utilization and related provision setting for apheresis** | | | | | | | |
| 11 | Pr_SC_processing_for_CTX_plus_G_CSF_w_wo_PLX_APH | Parameters (L44) | Probability of SC processing for patients on CTX 4 g/m^2^ + G-CSF ± on-demand PLX | 1.000 | 1.000 | 1.000 | Oncohematologists’ opinion |
| 12 | Pr_SC_freezing_for_CTX_plus_G_CSF_w_wo_PLX_APH | Parameters (L45) | Probability of SC cryopreservation for patients on CTX 4 g/m^2^ + G-CSF ± on-demand PLX | 1.000 | 1.000 | 1.000 | Oncohematologists’ opinion |
| 13 | Pr_SC_thawing_for_CTX_plus_G_CSF_w_wo_PLX_APH | Parameters (L46) | Probability of SC thawing for patients on CTX 4 g/m^2^ + G-CSF ± on-demand PLX | 1.000 | 1.000 | 1.000 | Oncohematologists’ opinion |
| 14 | Pr_Outpatient_FC_and_Full_Blood_Count_Chemo_free_APH | Parameters (L48) | Probability of outpatient flow cytometry for patients on G-CSF ± on-demand PLX | 1.000 | 1.000 | 1.000 | Oncohematologists’ opinion |
| 15 | Pr_SC_processing_for_Chemo_free_APH | Parameters (L50) | Probability of SC processing for patients on G-CSF ± on-demand PLX | 1.000 | 1.000 | 1.000 | Oncohematologists’ opinion |
| 16 | Pr_SC_freezing_for_Chemo_free_APH | Parameters (L51) | Probability of SC cryopreservation for patients on G-CSF ± on-demand PLX | 1.000 | 1.000 | 1.000 | Oncohematologists’ opinion |
| 17 | Pr_SC_thawing_for_Chemo_free_APH | Parameters (L52) | Probability of SC thawing for patients on G-CSF ± on-demand PLX | 1.000 | 1.000 | 1.000 | Oncohematologists’ opinion |

*APH* apheresis, *FC* flow cytometry, *CTX* cyclophosphamide, *CVC* central venous catheter, *DH* day-hospital, *G-CSF* granulocyte colony-stimulating factor; *Inw Hosp* inward hospitalization, *LL* lower limit, *MOB* mobilization, *PLX* plerixafor, *Pr* probability, *SC* stem cells, *UL* upper limit, *w* with, *wo* without.

## Supplementary Table S2 Parameters with no statistical distribution for base case and sensitivity analyses (one-way and probabilistic)

| **Parameter number** | **Parameter**  **name** | **Spreadsheet**  **(point estimate cell)** | **Parameter**  **description** | **Point estimate** | **Range** | | **Source** |
| --- | --- | --- | --- | --- | --- | --- | --- |
|  |  |  |  |  | **LL** | **UL** |  |
| **G-CSF alone vs CTX 4 g/m^2^ + G-CSF (± on demand PLX)** | | | | | | | |
| **Non healthcare resources: out-of-pocket expenses and caregiving – probability of utilization** | | | | | | |  |
| 18 | Pr_Car_Transportation_for_CTX_plus_G_CSF_w_wo_PLX | Parameters (L54) | Probability of car transportation for patients on CTX 4 g/m^2^ + G-CSF ± on-demand PLX | 1.000 | 1.000 | 1.000 | Oncohematologists’ opinion |
| 19 | Pr_Car_Transportation_for_Chemo_free | Parameters (L55) | Probability of car transportation for patients on G-CSF ± on-demand PLX | 1.000 | 1.000 | 1.000 | Oncohematologists’ opinion |
| 20 | Pr_care_giving_for_CTX_plus_G_CSF_w_wo_PLX | Parameters (L57) | Probability of caregiving for patients on CTX 4 g/m^2^ + G-CSF ± on-demand PLX | 1.000 | 1.000 | 1.000 | Oncohematologists’ opinion |
| 21 | Pr_care_giving_for_Chemo_free | Parameters (L58) | Probability of caregiving for patients on G-CSF ± on-demand PLX | 1.000 | 1.000 | 1.000 | Oncohematologists’ opinion |
| **Health care resources – Volumes and related provision setting - Mobilization** | | | | | | | |
| 22 | Daily_dose_CTX_for_CTX_plus_G_CSF_w_wo_PLX_MOB | Parameters (L74) | Daily dose of CTX for patients on CTX 4 g/m^2^ + G-CSF ± on-demand PLX | 4.000 | 4.000 | 4.000 | Oncohematologists’ opinion |
| 23 | Daily_dose_GCSF_for_CTX_plus_G_CSF_w_wo_PLX_MOB | Parameters (L76) | Daily dose of G-CSF (mcg/kg) for patients on CTX 4 g/m^2^ + G-CSF ± on-demand PLX | 5.000 | 5.000 | 5.000 | Oncohematologists’ opinion |
| 24 | Days_CTX_for_CTX_plus_G_CSF_w_wo_PLX_MOB | Parameters (L77) | Days of CTX administration for patients on CTX 4 g/m^2^ + G-CSF ± on-demand PLX | 1.000 | 1.000 | 1.000 | Oncohematologists’ opinion |
| 25 | Days_PLX_for_CTX_plus_G_CSF_w_wo_PLX_MOB | Parameters (L78) | Days of PLX administration for patients on CTX 4 g/m^2^ + G-CSF ± on-demand PLX | 1.000 | 1.000 | 1.000 | Oncohematologists’ opinion |
| 26 | Days_GCSF_for_CTX_plus_G_CSF_w_wo_PLX_MOB | Parameters (L79) | Days of G-CSF administration for patients on CTX 4 g/m^2^ + G-CSF ± on-demand PLX | 10.000 | 10.000 | 10.000 | Oncohematologists’ opinion |
| 27 | Days_PLX_for_Chemo_free_MOB | Parameters (L83) | Days of PLX administration for patients on G-CSF ± on demand PLX | 1.000 | 1.000 | 1.000 | Oncohematologists’ opinion |
| **Health care resources – Volumes and related provision setting - Apheresis** | | | | | | | |
| 28 | Vol_SC_processing_for_CTX_plus_G_CSF_w_wo_PLX_APH | Parameters (L96) | Number of procedure for SC processing for patients on CTX 4 g/m^2^ + G-CSF ± on-demand PLX | 1.000 | 1.000 | 1.000 | Oncohematologists’ opinion |
| 29 | Vol_SC_freezing_for_CTX_plus_G_CSF_w_wo_PLX_APH | Parameters (L97) | Number of procedure for SC cryopreservation for patients on CTX 4 g/m^2^ + G-CSF ± on-demand PLX | 1.000 | 1.000 | 1.000 | Oncohematologists’ opinion |
| 30 | Vol_SC_thawing_for_CTX_plus_G_CSF_w_wo_PLX_APH | Parameters (L98) | Number of procedure for SC thawing for patients on CTX 4 g/m^2^ + G-CSF ± on-demand PLX | 1.000 | 1.000 | 1.000 | Oncohematologists’ opinion |
| 31 | Vol_SC_processing_for_Chemo_free_APH | Parameters (L100) | Number of procedures for SC processing for patients on + G-CSF ± on-demand PLX | 1.000 | 1.000 | 1.000 | Oncohematologists’ opinion |
| 32 | Vol_SC_freezing_for_Chemo_free_APH | Parameters (L101) | Number of procedures for SC cryopreservation for patients on + G-CSF ± on-demand PLX | 1.000 | 1.000 | 1.000 | Oncohematologists’ opinion |
| 33 | Vol_SC_thawing_for_Chemo_free_APH | Parameters (L102) | Number of procedures for SC thawing for patients on + G-CSF ± on-demand PLX | 1.000 | 1.000 | 1.000 | Oncohematologists’ opinion |
| **Healthcare resources: Unit costs (€2019)** | | | | | | | |
| 34 | uc_INHS_funded_g_CTX | Parameters (L163) | Price per gram of CTX | €17.70 | €17.70 | €17.70 | ^2^ |
| 35 | uc_INHS_funded_vial_PLX | Parameters (L164) | Price per vial of PLX | €5098.62 | €5098.62 | €5098.62 | ^2^ |
| 36 | uc_INHS_funded_mg_GCSF | Parameters (L165) | Price per microgram of CTX | €0.32 | €0.32 | €0.32 | ^2^ |

*APH* apheresis, *CTX* cyclophosphamide, *G-CSF* granulocyte colony-stimulating factor, *INHS* Italian National Health Service, *LL* lower limit, *MOB* mobilization, *PLX* plerixafor, *Pr* probability, *SC* stem cell, *uc* unit cost, *UL* upper limit, *Vol* volume, *w* with, *wo* without.

## Supplementary Table S3 Parameters with statistical distribution for base case and sensitivity analyses (one-way and probabilistic)

| **Parameter number** | **Parameter name** | **Spreadsheet**  **(point estimate cell)** | **Parameter description** | **Observation** | **Mean** | **SE** | **95% CI** | | **Beta distribution parameters^a^** | | **Source** |
| --- | --- | --- | --- | --- | --- | --- | --- | --- | --- | --- | --- |
|  |  |  |  |  |  |  | **LL** | **UL** | **Alfa** | **Beta** |  |
| **G-CSF alone vs CTX 4 g/m^2^ + G-CSF (± on-demand PLX)** | | | | | | | | | | | |
| **Healthcare resources: probability of utilization for mobilization** | | | | | | | | | | | |
| 37 | Pr_PLX_for_CTX_plus_G_CSF_w_wo_PLX_MOB | Parameters (L23) | Probability of PLX for patients on CTX 4 g/m^2^ + G-CSF ± on-demand PLX | 711 ^b,c^ | 0.090 | 0.011 | 0.070 | 0.112 | 64 | 647 | ^3^ |
| 38 | Pr_No_PLX_for_CTX_plus_G_CSF_w_wo_PLX_MOB | Parameters (L24) | Probability of no PLX for patients on CTX 4 g/m^2^ + G-CSF ± on-demand PLX | 711 ^b,c^ | 0.910 | 0.011 | 0.888 | 0.930 | 647 | 64 | ^3^ |
| 39 | Pr_FebNeu_for_CTX_plus_G_CSF_w_wo_PLX_MOB | Parameters  (L25) | Probability of febrile neutropenia for patients on CTX 4 g/m^2^ + G-CSF ± on-demand PLX | 711 ^b,c^ | 0.144 | 0.013 | 0.119 | 0.171 | 102 | 609 | Onco-hematologists’ opinion |
| 40 | Pr_cond_Inw_Hosp_FebNeu_for_CTX_plus_G_CSF_w_wo_PLX_MOB | Parameters (L26) | Conditional probability of inward hospitalization given febrile neutropenia for patients on CTX 4 g/m^2^ + G-CSF ± on-demand PLX | 102 ^c,d^ | 0.800 | 0.040 | 0.718 | 0.871 | 82 | 20 | Oncohematologists’ opinion |
| 41 | Pr_RBC_transfusion_for_CTX_plus_G_CSF_w_wo_PLX_MOB | Parameters (L32) | Probability of RBC transfusions for patients on CTX 4 g/m^2^ + G-CSF ± on-demand PLX | 711 ^b,c^ | 0.120 | 0.012 | 0.097 | 0.145 | 85 | 626 | Onco-hematologists’ opinion |
| 42 | Pr_PLT_transfusion_for_CTX_plus_G_CSF_w_wo_PLX_MOB | Parameters (L33) | Probability of PLT transfusions for patients on CTX 4 g/m^2^ + G-CSF ± on-demand PLX | 711 ^b,c^ | 0.120 | 0.012 | 0.097 | 0.145 | 85 | 626 | Onco-hematologists’ opinion |
| 43 | Pr_PLX_for_Chemo_free_MOB | Parameters (L35) | Probability of PLX for patients on G-CSF ± on-demand PLX | 70 ^c,e^ | 0.470 | 0.060 | 0.355 | 0.587 | 33 | 37 | Onco-hematologists’ opinion |
| 44 | Pr_No_PLX_for_Chemo_free_MOB | Parameters (L36) | Probability of no PLX for patients on G-CSF ± on-demand PLX | 70 ^c,e^ | 0.530 | 0.060 | 0.413 | 0.645 | 37 | 33 | Onco-hematologists’ opinion |

*CI* confidence interval, *cond* conditional, *CTX* cyclophosphamide, *FebNeu* febrile neutropenia, *G-CSF* granulocyte colony-stimulating factor, *Inw Hosp* inward hospitalization, *LL* lower limit, *MM* multiple myeloma, *MOB* mobilization, *PLT* platelet, *PLX* plerixafor, *Pr* probability, *RBC* red blood cell, *SE* standard error, *UL* upper limit, *w* with, *wo* without.

^a^A beta distribution was fitted to binomial data (e.g,, two-level categorical parameters, such as receiving on-demand PLX or not) ^4, 5^.

^b^Number of MM patients mobilized with CTX 4 g/m2 + G-CSF (± on-demand PLX) by the oncohematologic centers of Catania, Florence, Milan, Ravenna, and Rome during 2013–2017 ^3^.

^c^The number of observations does not affect parameter sample estimate but SE and 95% CI only.

^d^Number of MM patients with febrile neutropenia out of 711 MM patients mobilized with CTX 4 g/m2 + G-CSF (± on-demand PLX) by the oncohematologic centers of Catania, Florence, Milan, Ravenna, and Rome during 2013–2017 ^3^ .

^e^Number of MM patients mobilized with G-CSF (± on-demand PLX) by the oncohematologic centers of Catania, Florence, Milan, Ravenna, and Rome during 2013-2017 ^3^.

**Supplementary Table S4** Parameters with statistical distribution for base case and sensitivity analyses (one-way and probabilistic)

| **Parameter number** | **Parameter name** | **Spreadsheet**  **(point estimate cell)** | **Parameter description** | **Observations** | **Mean** | **SE** | **95% CI** | | **Beta distribution parameters^a^** | | **Source** |
| --- | --- | --- | --- | --- | --- | --- | --- | --- | --- | --- | --- |
|  |  |  |  |  |  |  | **LL** | **UL** | **Alfa** | **Beta** |  |
| **G-CSF alone vs CTX 4 g/m^2^ + G-CSF (± on-demand PLX)** | | | | | | | | | | | |
| **Health care resources – Probability of consumption - Apheresis** | | | | | | | | | | | |
| 45 | Pr_Inw_Hosp_FC_and_Full_Blood_Count_CTX_plus_G_CSF_w_wo_PLX_APH | Parameters (L41) | Probability of flow cytometry and full blood count in inpatient setting for patients on CTX 4 g/m^2^ + G-CSF ± on-demand PLX | 711 ^b,c^ | 0.115 | 0.012 | 0.093 | 0.139 | 82 | 629 | Onco-hematologists’ opinion |
| 46 | Pr_Outpatient_FC_and_Full_Blood_Count_CTX_plus_G_CSF_w_wo_PLX_APH | Parameters (L42) | Probability of flow cytometry and full blood count in outpatient setting for patients on CTX 4 g/m^2^ + G-CSF ± on-demand PLX | 711 ^b,c^ | 0.885 | 0.012 | 0.861 | 0.907 | 629 | 82 | Onco-hematologists’ opinion |
| **Non health care resources – Productivity losses - Probability of occurrence** | | | | | | | | | | | |
| 47 | Pr_employed_for_CTX_plus_G_CSF_w_wo_PLX | Parameters (L58) | Probability of being employed for patients on CTX 4 g/m^2^ + G-CSF ± on-demand PLX | 711 ^b,c^ | 0.500 | 0.019 | 0.463 | 0.537 | 356 | 356 | Onco-hematologists’ opinion |
| 48 | Pr_employed_for_Chemo_free | Parameters (L59) | Probability of being employed for patients on G-CSF ± PLX on demand | 70 ^c,d^ | 0.500 | 0.060 | 0.384 | 0.616 | 35 | 35 | Onco-hematologists’ opinion |

*APH* apheresis, *CI* confidence interval, *CTX* cyclophosphamide, *FC* flow cytometry, *G-CSF* granulocyte colony-stimulating factor; *Inw Hosp* inward hospitalization, *LL* lower limit, *MM* multiple myeloma, *PLX* plerixafor, *Pr* probability, *SE* standard error, *UL* upper limit, *w* with, *wo* without.

^a^A beta distribution was fitted to binomial data (e.g., two-level categorical parameters, such as receiving on-demand PLX or not) ^4, 5^.

^b^Number of MM patients mobilized with CTX 4 g/m2 + G-CSF (±on-demand PLX chemotherapy) by the oncohematologic centers of Catania, Florence, Milan, Ravenna, and Rome during 2013-2017 ^3^.

^c^The number of observations does not affect parameter sample estimate but SE and 95% CI only.

^d^Number of MM patients mobilized with G-CSF (±on-demand PLX) by the oncohematologic centers of Catania, Florence, Milan, Ravenna, and Rome during 2013-2017 ^3^.

## Supplementary Table S5 Parameters with statistical distribution for base case and sensitivity analyses (one-way and probabilistic)

| **Parameter number** | **Parameter name** | **Spreadsheet**  **(point estimate cell)** | **Parameter description** | **Observations** | **Mean** | **SE** | **95% CI** | | **Beta distribution parameters^a^** | | | | **Source** |
| --- | --- | --- | --- | --- | --- | --- | --- | --- | --- | --- | --- | --- | --- |
|  |  |  |  |  |  |  | **LL** | **UL** | **Alfa** | | | **Beta** |  |
| **G-CSF alone vs CTX 4 g/m^2^ + G-CSF (± on-demand PLX)** | | | | | | | | | | | | | |
| **Effectiveness: probability of successful 4x10^6^ CD34^+^ apheresis** | | | | | | | | | | | | | |
| 49 | CTX_plus_G_CSF_wo_PLX_EFF | Parameters (L151) | Probability of successful 4x10^6^ CD34+ apheresis for patients on CTX 4 g/m^2^ + G-CSF without on-demand PLX | 647^b,c^ | 0.702 | 0.018 | 0.666 | 0.737 | | 454 | 193 | | ^1^, onco-hematologists’ opinion |
| 50 | CTX_plus_G_CSF_w_PLX_EFF | Parameters (L152) | Probability of successful 4x10^6^ CD34+ apheresis for patients on CTX 4 g/m^2^ + G-CSF with on-demand PLX | 64^b,c^ | 0.838 | 0.046 | 0.739 | 0.917 | | 54 | 10 | | ^1^, onco-hematologists’ opinion |
| 51 | Chemo_free_wo_PLX_EFF | Parameters (L154) | Probability of successful 4x10^6^ CD34+ apheresis for patients on G-CSF without on-demand PLX | 37 ^c,d^ | 0.702 | 0.075 | 0.548 | 0.836 | | 26 | 11 | | ^1^, onco hematologists’ opinion |
| 52 | Chemo_free_w_PLX_EFF | Parameters (L155) | Probability of successful 4x10^6^ CD34+ apheresis for patients on G-CSF with on-demand PLX | 33 ^c,d^ | 0.838 | 0.064 | 0.697 | 0.941 | | 28 | 5 | | ^1^, onco-hematologists’ opinion |

*CI* confidence interval, *CTX* cyclophosphamide, *EFF* effectiveness, *G-CSF* granulocyte colony-stimulating factor, *LL* lower limit, *MM* multiple myeloma, *PLX* plerixafor, *Pr* probability, *SE* standard error, *UL* upper limit, *w* with, *wo* without.

^a^A beta distribution was fitted to binomial data (e.g., two-level categorical parameters, such as receiving on-demand PLX or not) ^4, 5^.

^b^Out of 711 MM patients mobilized (± chemotherapy) by the oncohematologic centers of Catania, Florence, Milan, Ravenna, and Rome during 2013-2017 ^3^.

^c^The number of observations does not affect parameter sample estimate but SE and 95% CI only.

^d^Out of 70 MM patients mobilized with G-CSF (± on-demand PLX) by the oncohematologic centers of Catania, Florence, Milan, Ravenna, and Rome during 2013-2017 ^3^.

## Supplementary Table S6 Parameters with statistical distribution for base case and sensitivity analyses (one-way and probabilistic)

| **Parameter number** | **Parameter**  **name** | **Spreadsheet**  **(point estimate cell)** | **Parameter**  **description** | **Mean** | **SE** | **95% CI** | | **Gamma distribution parameters^a^** | | **Source** |
| --- | --- | --- | --- | --- | --- | --- | --- | --- | --- | --- |
|  |  |  |  |  |  | **LL** | **UL** | **Alfa** | **Beta** |  |
| **G-CSF alone vs CTX 4 g/m^2^ + G-CSF (± on-demand PLX)** | | | | | | | | | | |
| **Anthropometric parameters** | | | | | | | | | | |
| 53 | BW_kg_CTX_plus_G_CSF_w_wo_PLX | Parameters (L14) | Body weight patients on CTX 4 g/m^2^ + G-CSF ± on-demand PLX | 70.00 | 28.00 ^b^ | 26.34 | 134.61 | 6 | 11 | Onco-hematologists’ opinion |
| 54 | BW_kg_Chemo_free | Parameters (L15) | Body weight patients on G-CSF ± on-demand PLX | 70.00 | 28.00 ^b^ | 26.34 | 134.61 | 6 | 11 | Onco-hematologists’ opinion |
| **Health care resources – Volume - Mobilization** | | | | | | | | | | |
| 55 | Vol_Full_Blood_Count_for_CTX_plus_G_CSF_w_wo_PLX_MOB | Parameters (L63) | Number of full blood count for patients on CTX 4 g/m^2^ + G-CSF ± on-demand PLX | 1.50 | 0.60 ^b^ | 0.57 | 2.89 | 6 | 0.24 | Onco-hematologists’ opinion |
| 56 | Vol_Full_Blood_Count_for_Chemo_free_MOB | Parameters (L68) | Number of full blood count for patients on G-CSF ± on-demand PLX | 1.50 | 0.60 ^b^ | 0.57 | 2.89 | 6 | 0.24 | Onco-hematologists’ opinion |
| 57 | Vol_CVC_for_CTX_plus_G_CSF_w_wo_PLX_MOB | Parameters (L73) | Number of central venous catheter for patients on CTX 4 g/m^2^ + G-CSF ± on-demand PLX | 1.00 | 0.40 ^b^ | 0.38 | 1.92 | 6 | 0.16 | Onco-hematologists’ opinion |
| 58 | Daily_dose_PLX_for_CTX_plus_G_CSF_w_wo_PLX_MOB | Parameters (L75) | Number of PLX vials for patients on CTX 4 g/m^2^ + G-CSF ± on-demand PLX | 1.50 | 0.60 ^b^ | 0.57 | 2.89 | 6 | 0.24 | Onco-hematologists’ opinion |
| 59 | Days_GCSF_for_CTX_plus_G_CSF_w_wo_PLX_MOB | Parameters (L79) | Days of G-CSF administration for patients on CTX 4 g/m^2^ + G-CSF ± on-demand PLX | 10.00 | 4.00 ^b^ | 3.76 | 19.23 | 6 | 1.60 | Onco-hematologists’ opinion |
| 60 | Daily_dose_PLX_for_Chemo_free_MOB | Parameters (L81) | Number of PLX vials for patients on G-CSF ± on-demand PLX | 1.50 | 0.60 ^b^ | 0.57 | 2.89 | 6 | 0.24 | Onco-hematologists’ opinion |
| 61 | Days_GCSF_for_Chemo_free_MOB | Parameters (L84) | Days of G-CSF administration for patients on G-CSF ± on-demand PLX | 6.00 | 2.40 ^b^ | 2.26 | 11.54 | 6 | 0.96 | Onco-hematologists’ opinion |
| 62 | Vol_RBC_transf_for_CTX_plus_G_CSF_w_wo_PLX_MOB | Parameters (L86) | Number of RBC transfusions for patients on CTX 4 g/m^2^ + G-CSF ± on-demand PLX | 2. 00 | 0.80 ^b^ | 0.75 | 3.85 | 6 | 0.32 | Onco-hematologists’ opinion |
| 63 | Vol_PLT_transf_for_CTX_plus_G_CSF_w_wo_PLX_MOB | Parameters (L87) | Number of PLT transfusions for patients on CTX 4 g/m^2^ + G-CSF ± on-demand PLX | 1.00 | 0.40 ^b^ | 0.38 | 1.92 | 6 | 0.16 | Onco-hematologists’ opinion |
| 64 | Days_Inw_Hosp_CTX_CTX_plus_G_CSF_w_wo_PLX_MOB | Parameters (L89) | Days of hospitalization for CTX administration for patients on CTX 4 g/m^2^ + G-CSF ± on-demand PLX | 2.00 | 0.80 ^b^ | 0.75 | 3.85 | 6 | 0.32 | Onco-hematologists’ opinion |
| 65 | DH_Access_PLX_CTX_plus_G_CSF_w_wo_PLX_MOB | Parameters (L90) | Number of day-hospital attendances for PLX administration for patients on CTX 4 g/m^2^ + G-CSF ± on-demand PLX | 1.00 | 0.40 ^b^ | 0.38 | 1.92 | 6 | 0.16 | Onco-hematologists’ opinion |
| 66 | Days_Inw_Hosp_FebNeu_for_CTX_plus_G_CSF_w_wo_PLX_MOB | Parameters (L91) | Days of hospitalization for febrile neutropenia for patients on CTX 4 g/m^2^ + G-CSF ± on-demand PLX | 3.00 | 1.20 ^b^ | 1.13 | 5.77 | 6 | 0.48 | Onco-hematologists’ opinion |
| 67 | DH_Access_PLX_Chemo_free | Parameters (L94) | Number of day-hospital attendances for PLX administration for patients on G-CSF ± on-demand PLX | 1.00 | 0.40 ^b^ | 0.38 | 1.92 | 6 | 0.16 | Onco-hematologists’ opinion |

*BW* body weight, *CI* confidence interval, *CTX* cyclophosphamide, *CV* coefficient of variation, *CVC* central venous catheter, *DH* day-hospital, *G-CSF* granulocyte colony-stimulating factor, *Inw Hosp* inward hospitalization, *LL* lower limit, *MOB* mobilization, *PLX* plerixafor, *PLT* platelet, *RBC* red blood cell, *SE* standard error, *transf* transfusion, *UL* upper limit, *Vol* volume, *w* with, *wo* without.

^a^ A gamma distribution was used to represent uncertainty in anthropometric parameters (e.g., patients’ BW) and resource consumption (e.g., number of PLX vials) ^4, 5^.

^b^If the analytical calculation of SE was unfeasible, a CV of 40% of the sample mean was imposed ^4-6^.

## Supplementary Table S7 Parameters with statistical distribution for base case and sensitivity analyses (one-way and probabilistic)

| **Parameter number** | **Parameter name** | **Spreadsheet**  **(point estimate cell)** | **Parameter description** | | **Mean** | **SE** | **95% CI** | | | **Gamma distribution parameters^a^** | | **Source** |
| --- | --- | --- | --- | --- | --- | --- | --- | --- | --- | --- | --- | --- |
|  |  |  |  |  |  |  | **LL** | **UL** | **Alfa** | | **Beta** |  |
| **G-CSF alone vs CTX 4 g/m^2^ + G-CSF (± on-demand PLX)** | | | | | | | | | | | | |
| **Healthcare resources: Apheresis (volume)** | | | | | | | | | | | | |
| 68 | Vol_FC_and_Full_Blood_Count_CTX_plus_G_CSF_w_wo_PLX_APH | Parameters (L64) | Number of flow cytometries and full blood counts for patients on CTX 4 g/m^2^ + G-CSF ± on-demand PLX | | 1.50 | 0.60 ^b^ | 0.57 | 2.89 | 6 | | 0.24 | Onco-hematologists’ opinion |
| 69 | Vol_Apheresis_for_CTX_plus_G_CSF_w_wo_PLX_APH | Parameters (L65) | Number of aphereses for patients on CTX 4 g/m^2^ + G-CSF ± on-demand PLX | | 1.60 | 0.64 ^b^ | 0.60 | 3.08 | 6 | | 0.26 | Onco-hematologists’ opinion |
| 70 | Vol_Missed_APH_CTX_plus_G_CSF_w_wo_PLX_APH | Parameters (L66) | Number of missed apheresis due to poor mobilization for patients on CTX 4 g/m^2^ + G-CSF ± on-demand PLX | | 0.95 | 0.38 ^b^ | 0.36 | 1.83 | 6 | | 0.15 | Onco-hematologists’ opinion |
| 71 | Vol_FC_and_Full_Blood_Count_Chemo_free_APH | Parameters (L69) | Number of flow cytometries and full blood counts for patients G-CSF ± on-demand PLX | | 1.50 | 0.60 ^b^ | 0.57 | 2.89 | 6 | | 0.24 | Onco-hematologists’ opinion |
| 72 | Vol_Apheresis_for_Chemo_free_APH | Parameters (L70) | Number of apheresis sessions for patients on G-CSF ± PLX on demand | | 2.00 | 0.80 ^b^ | 0.75 | 3.85 | 6 | | 0.32 | Onco-hematologists’ opinion |
| 73 | Vol_Missed_APH_Chemo_free_APH | Parameters (L71) | Number of missed apheresis sessions due to poor mobilization for patients on G-CSF ± on-demand PLX | | 0.48 | 0.19 ^b^ | 0.18 | 0.92 | 6 | | 0.08 | Onco-hematologists’ opinion |
| **Non-healthcare resources: informal care (volume)** | | | |  | | | | | | | | |
| 74 | Vol_caregivers_CTX_plus_G_CSF_w_wo_PLX | Parameters (L106) | Number of caregivers for patients on CTX 4 g/m^2^ + G-CSF ± on-demand PLX | | 0.84 | 0.34 ^b^ | 0.32 | 1.61 | 6 | | 0.13 | Onco-hematologists’ opinion |
| 75 | Vol_caregivers_Chemo_free | Parameters (L107) | Number of caregivers for patients on G-CSF ± on-demand PLX | | 0.86 | 0.34 ^b^ | 0.32 | 1.65 | 6 | | 0.14 | Onco-hematologists’ opinion |
| **Non-healthcare resources: out-of-pocket expenses (volume)** | | | |  | | | | | | | | |
| 76 | Km_from_home_to_go_CTX_plus_G_CSF_w_wo_PLX | Parameters (L109) | Distance in km from home to healthcare facility (one-way only) for patients on CTX 4 g/m^2^ + G-CSF ± on-demand PLX | | 52.77 | 21.11 ^b^ | 19.86 | 101.48 | 6 | | 8 | Onco-hematologists’ opinion |
| 77 | Km_from_home_to_go_Chemo_free | Parameters (L110) | Distance in km from home to healthcare facility (one-way only) for patients on G-CSF ± on-demand PLX | | 50.13 | 20.05 ^b^ | 18.87 | 96.41 | 6 | | 8 | Onco-hematologists’ opinion |
| 78 | Minutes_Transportation_CTX_plus_G_CSF_w_wo_PLX | Parameters (L111) | Minutes of transportation from home to healthcare facility (one-way only) for patients on CTX 4 g/m^2^ + G-CSF ± on-demand PLX | | 63.33 | 25.33 ^b^ | 23.83 | 121.78 | 6 | | 10 | Research assumption |
| 79 | Minutes_Transportation_Chemo_free | Parameters (L112) | Minutes of transportation from home to healthcare facility (one-way only) for patients on G-CSF ± on-demand PLX | | 60.16 | 24.06 ^b^ | 22.64 | 115.69 | 6 | | 10 | Research assumption |

*APH* apheresis, *CI* confidence interval, *CTX* cyclophosphamide, *CV* coefficient of variation, *FC* flow cytometry, *G-CSF* granulocyte colony-stimulating factor; *LL* lower limit, *MOB* mobilization, *PLX* plerixafor, *Pt* patient, *SE* standard error, *UL* upper limit, *Vol* volume, *w* with, *wo* without.

^a^ A gamma distribution was used to represent uncertainty in resource consumption (e.g., number of APH sessions) ^4, 5^.

^b^If the analytical calculation of SE was unfeasible, a CV of 40% of the sample mean was imposed^4-6^.

## Supplementary Table S8 Parameters with statistical distribution for base case and sensitivity analyses (one-way and probabilistic)

| **Parameter number** | **Parameter name** | **Spreadsheet**  **(point estimate cell)** | **Parameter description** | **Mean** | | **SE** | **95% CI** | | **Gamma distribution parameters^a^** | | **Source** |
| --- | --- | --- | --- | --- | --- | --- | --- | --- | --- | --- | --- |
|  |  |  |  |  |  |  | **LL** | **UL** | **Alfa** | **Beta** |  |
| **G-CSF alone vs CTX 4 g/m^2^ + G-CSF (± on-demand PLX)** | | | | | | | | | | | |
| **Non-healthcare resources: out-of-pocket expenses (volume)** | | | | | | | | | | | |
| 80 | Pkg_min_CTX_plus_G_CSF_w_wo_PLX_MOB | Parameters (L114) | Daily minutes of hospital parking (CTX administration) for pts on CTX 4 g/m^2^ + G-CSF ± on-demand PLX | 480.00 | 192.00 ^b^ | | 180.64 | 923.07 | 6 | 77 | Research assumption |
| 81 | Pkg_min_DH_PLX_for_CTX_plus_G_CSF_w_wo_PLX_MOB | Parameters (L115) | Daily minutes of hospital parking (PLX administration) for pts on CTX 4 g/m^2^ + G-CSF ± on-demand PLX | 300.00 | 120.00 ^b^ | | 112.90 | 576.92 | 6 | 48 | Research assumption |
| 82 | Pkg_min_Inw_Hosp_FebNeu_for_CTX_plus_G_CSF_w_wo_PLX_MOB | Parameters (L116) | Daily minutes of hospital parking (febrile neutropenia) for pts on CTX 4 g/m^2^ + G-CSF ± on-demand PLX | 480.00 | 192.00 ^b^ | | 180.64 | 923.07 | 6 | 77 | Research assumption |
| 83 | Pkg_min_Inw_Hosp_FC_and_BlTe_CTX_plus_G_CSF_w_wo_PLX_APH | Parameters (L117) | Daily minutes of hospital parking (inpatient apheresis) for pts on CTX 4 g/m^2^ + G-CSF ± on-demand PLX | 480.00 | 192.00 ^b^ | | 180.64 | 923.07 | 6 | 77 | Research assumption |
| 84 | Pkg_min_Outpatient_FC_and_BlTe_CTX_plus_G_CSF_w_wo_PLX_APH | Parameters (L118) | Daily minutes of hospital parking (outpatient apheresis) for pts on CTX 4 g/m^2^ + G-CSF ± on-demand PLX | 300.00 | 120.00 ^b^ | | 112.90 | 576.92 | 6 | 48 | Research assumption |
| 85 | Pkg_min_DH_PLX_Chemo_free_MOB | Parameters (L120) | Daily minutes of hospital parking (PLX administration) for pts on G-CSF without on-demand PLX | 300.00 | 120.00 ^b^ | | 112.90 | 576.92 | 6 | 48 | Research assumption |
| 86 | Pkg_min_Inw_Hosp_FC_and_BlTe_Chemo_free_APH | Parameters (L121) | Daily minutes of hospital parking (febrile neutropenia) for pts on G-CSF without on-demand PLX | 480.00 | 192.00 ^b^ | | 180.64 | 923.07 | 6 | 77 | Research assumption |
| 87 | Pkg_min_Outpatient_FC_and_BlTe_Chemo_free_APH | Parameters (L122) | Daily minutes of hospital parking (outpatient apheresis) for pts on G-CSF ± on-demand PLX | 300.00 | 120.00 ^b^ | | 112.90 | 576.92 | 6 | 48 | Research assumption |

*BlTe* blood tests, *CI* confidence interval, *CTX* cyclophosphamide, *CV* coefficient of variation, *DH* day hospital, *FC* flow cytometry, *FebNeu* febrile neutropenia, *G-CSF* granulocyte colony-stimulating factor, *Inw Hosp* inward hospitalization, *LL* lower limit, *MOB* mobilization, *pkg* parking, *PLX* plerixafor, *pt* patient, *SE* standard error, *UL* upper limit, *w* with, *wo* without.

^a^ A gamma distribution was used to represent uncertainty in resource consumption (e.g., daily minutes of hospital pkg) ^4, 5^.

^b^If the analytical calculation of SE was unfeasible, a CV of 40% of the sample mean was imposed ^4-6^.

## Supplementary Table S9 Parameters with statistical distribution for base-case and sensitivity analyses (one-way and probabilistic)

| **Parameter number** | **Parameter name** | **Spreadsheet**  **(point estimate cell)** | **Parameter description** | **Mean** | **SE** | **95% CI** | | **Gamma distribution parameters^a^** | | **Source** |
| --- | --- | --- | --- | --- | --- | --- | --- | --- | --- | --- |
|  |  |  |  |  |  | **LL** | **UL** | **Alfa** | **Beta** |  |
| **G-CSF alone vs CTX 4 g/m^2^ + G-CSF (± on-demand PLX)** | | | | | | | | | | |
| **Non-healthcare resources: patient’s time (volume)** | | | | | | | | | | |
| 88 | Pt_min_CTX_plus_G_CSF_w_wo_PLX_MOB | Parameters (L126) | Daily min spent at hospital (CTX administration) for pts on CTX 4 g/m^2^ + G-CSF ± on-demand PLX | 480.00 | 192.00 ^b^ | 180.64 | 923.07 | 6 | 10 | Research assumption |
| 89 | Pt_min_Inw_Hosp_FebNeu_for_CTX_plus_G_CSF_w_wo_PLX_MOB | Parameters (L127) | Daily min spent at hospital (febrile neutropenia) for pts on CTX 4 g/m^2^ + G-CSF ± on-demand PLX | 480.00 | 192.00 ^b^ | 180.64 | 923.07 | 6 | 10 | Research assumption |
| 90 | Pt_min_DH_PLX_CTX_plus_G_CSF_w_wo_PLX_MOB | Parameters (L128) | Daily min spent at hospital (PLX administration) for pts on CTX 4 g/m^2^ + G-CSF ± on-demand PLX | 300.00 | 120.00 ^b^ | 112.90 | 576.92 | 6 | 48 | Research assumption |
| 91 | Pt_min_Inw_Hosp_FC_and_BlTe_CTX_plus_G_CSF_w_wo_PLX_APH | Parameters (L129) | Daily min spent at hospital (inward apheresis) for pts on CTX 4 g/m^2^ + G-CSF ± on-demand PLX | 480.00 | 19.20 ^b^ | 180.64 | 923.07 | 6 | 77 | Research assumption |
| 92 | Pt_min_Outpatient_FC_and_BlTe_for_CTX_plus_G_CSF_w_wo_PLX_APH | Parameters (L130) | Daily min spent at hospital (outpatient apheresis) for pts on CTX 4 g/m^2^ + G-CSF ± on-demand PLX | 300.00 | 120.00 ^b^ | 112.90 | 576.92 | 6 | 48 | Research assumption |
| 93 | Pt_min_DH_PLX_Chemo_free_MOB | Parameters (L132) | Daily min spent at hospital (PLX administration) for pts on G-CSF ± on-demand PLX | 300.00 | 120.00 ^b^ | 112.90 | 576.92 | 6 | 48 | Research assumption |
| 94 | Pt_min_Inw_Hosp_FC_and_BlTe_Chemo_free_APH | Parameters (L133) | Daily min spent at hospital (inpatient apheresis) for pts on G-CSF ± on-demand PLX | 480.00 | 192.00 ^b^ | 180.64 | 923.07 | 6 | 77 | Research assumption |
| 95 | Pt_min_Outpatient_FC_and_BlTe_Chemo_free_APH | Parameters (L134) | Daily min spent at hospital (outpatient apheresis) for pts on G-CSF ± on-demand PLX | 300.00 | 120.00 ^b^ | 112.90 | 576.92 | 6 | 48 | Research assumption |

*BlTe* blood tests, CI confidence interval, *CTX* cyclophosphamide, *CV* coefficient of variation, *DH* day-hospital, *FC* flow cytometry, *FebNeu* febrile neutropenia, *G-CSF* granulocyte colony-stimulating factor, *Inw Hosp* inward hospitalization, *LL* lower limit, *MOB* mobilization, *PLX* plerixafor; *pt* patient, *SE* standard error, *UL* upper limit, *w* with, *wo* without.

^a^ A gamma distribution was used to represent uncertainty in resource consumption (e.g., daily minutes spent by pt in DH due to PLX administration) ^4, 5^.

^b^If the analytical calculation of SE was unfeasible, a CV of 40% of the sample mean was imposed ^4-6^.

## Supplementary Table S10 Parameters with statistical distribution for base case and sensitivity analyses (one-way and probabilistic)

| **Parameter number** | **Parameter name** | **Spreadsheet**  **(point estimate cell)** | **Parameter description** | **Mean** | | **SE** | **95% CI** | | **Gamma distribution parameters^a^** | | **Source** |
| --- | --- | --- | --- | --- | --- | --- | --- | --- | --- | --- | --- |
|  |  |  |  |  |  |  | **LL** | **UL** | **Alfa** | **Beta** |  |
| **G-CSF alone vs CTX 4 g/m^2^ + G-CSF (± on-demand PLX)** | | | | | | | | | | | |
| **Non-healthcare resources: informal care (volume)** | | | | | | | | | | | |
| 96 | CG_min_CTX_plus_G_CSF_w_wo_PLX_MOB | Parameters (L136) | Daily min spent by CG at hospital (CTX administration) for pts on CTX 4 g/m^2^ + G-CSF ± on-demand PLX | 402.15 | 160.86 ^b^ | | 151.35 | 773.36 | 6 | 64 | Research assumption |
| 97 | CG_min_Inw_Hosp_FebNeu_for_CTX_plus_G_CSF_w_wo_PLX_MOB | Parameters (L137) | Daily min spent by CG at hospital (febrile neutropenia) for pts on CTX 4 g/m^2^ + G-CSF ± on-demand PLX | 402.15 | 160.86 ^b^ | | 151.35 | 773.36 | 6 | 64 | Research assumption |
| 98 | CG_min_DH_PLX_CTX_plus_G_CSF_w_wo_PLX_MOB | Parameters (L138) | Daily min spent by CG at hospital (PLX administration) for pts on CTX 4 g/m^2^ + G-CSF ± on-demand PLX | 201.08 | 80.43 ^b^ | | 75.67 | 386.68 | 6 | 32 | Research assumption |
| 99 | CG_min_Inw_Hosp_FC_and_BlTe_CTX_plus_G_CSF_w_wo_PLX_APH | Parameters (L139) | Daily min spent by CG at hospital (inpatient apheresis) for pts on CTX 4 g/m^2^ + G-CSF ± on-demand PLX | 402.15 | 160.86 ^b^ | | 151.35 | 773.36 | 6 | 64 | Research assumption |
| 100 | CG_time_Outpatient_FC_and_BlTe_for_CTX_plus_G_CSF_w_wo_PLX_APH | Parameters (L140) | Daily min spent by CG at hospital (outpatient apheresis) for pts on CTX 4 g/m^2^ + G-CSF ±on-demand PLX | 201.08 | 80.43 ^b^ | | 75.67 | 386.68 | 6 | 32 | Research assumption |
| 101 | CG_min_DH_PLX_Chemo_free_MOB | Parameters (L142) | Daily min spent by CG at hospital (PLX administration) for pts on G-CSF ± on-demand PLX | 189.98 | 75.99 ^b^ | | 71.50 | 356.33 | 6 | 30 | Research assumption |
| 102 | CG_min_Inw_Hosp_FC_and_BlTe_Chemo_free_APH | Parameters (L143) | Daily min spent by CG at hospital (inpatient apheresis) for pts on G-CSF ± on-demand PLX | 379.95 | 151.98 ^b^ | | 142.99 | 730.67 | 6 | 61 | Research assumption |
| 103 | CG_min_Inw_Hosp_FC_and_BlTe_Chemo_free_APH | Parameters (L144) | Daily min spent by CG at hospital (outpatient apheresis) for pts on G-CSF ± on-demand PLX | 189.98 | 75.99 ^b^ | | 71.50 | 356.33 | 6 | 30 | Research assumption |

*APH* apheresis, *BlTe* blood tests, *CG* caregiver, *CI* confidence interval, *CTX* cyclophosphamide, *CV* coefficient of variation, *DH* day-hospital, *FC* flow cytometry, *FebNeu* febrile neutropenia, *G-CSF* granulocyte colony-stimulating factor, *Inw Hosp* inward hospitalization, *LL* lower limit, *MOB* mobilization, *PLX* plerixafor, *pt* patient, *SE* standard error, *UL* upper limit, *w* with, *wo* without.

^a^ A gamma distribution was used to represent uncertainty in resource consumption (e.g., daily minutes spent by CG in DH for pts due to PLX administration) ^4, 5^.

^b^If the analytical calculation of SE was unfeasible, a CV of 40% of the sample mean was imposed ^4-6^.

## Supplementary Table S11 Parameters with normal statistical distribution for base case and sensitivity analyses (one-way and probabilistic)

| **Parameter number** | **Parameter name** | **Spreadsheet**  **(point estimate cell)** | **Parameter description** | **Mean** | | **SE** | **95% CI** | | **Normal distribution parameters^a^** | | **Source** |  |
| --- | --- | --- | --- | --- | --- | --- | --- | --- | --- | --- | --- | --- |
|  |  |  |  |  |  |  | **LL** | **UL** | **Mean** | **SE** |  |  |
| **G-CSF alone vs CTX 4 g/m^2^ + G-CSF (± on-demand PLX)** | | | | | | | | | | | |  |
| **Healthcare resources: unit costs (€2019)** | | | | | | | | | | | | |
| 104 | uc_INHS_funded_Full_Blood_Count_MOB_APH | Parameters (L159) | Unit cost INHS-funded full blood count in outpatient setting (mobilization and apheresis) | €3.17 | €0.32^b^ | | €2.55 | €3.79 | €3.17 | €0.32 | ^7^ |  |
| 105 | uc_INHS_funded_FC_APH | Parameters (L160) | Unit cost INHS-funded flow cytometry in outpatient setting | €34.00 | €3.40 ^b^ | | €27.34 | €40.66 | €34.00 | €3.40 | ^7^ |  |
| 106 | uc_INHS_funded_APH_APH | Parameters (L161) | Unit cost INHS-funded apheresis in outpatient setting | €438.99 | €43.90 ^b^ | | €352.25 | €525.03 | €438.99 | €49.30 | ^7^ |  |
| 107 | uc_INHS_funded_CVC | Parameters (L166) | Unit cost INHS-funded CVC | €150.58 | €15.06 ^b^ | | €121. 07 | €180.09 | €150.58 | €15.06 | ^8^ |  |
| 108 | uc_INHS_funded_Days_CTX_for_CTX_plus_G_CSF_w_wo_PLX_MOB | Parameters (L168) | Unit cost INHS-funded day inward hospitalization (CTX administration) for pts on CTX 4 g/m^2^ + G-CSF ± on-demand PLX | €211.03 | €21.10 ^b^ | | €169.67 | €252.04 | €211.03 | €21.10 | ^9, 10^ |  |
| 109 | uc_INHS_funded_DH_Access_PLX_CTX_plus_G_CSF_w_wo_PLX_MOB | Parameters (L169) | Unit cost INHS-funded DH access (PLX administration) for pts on CTX 4 g/m^2^ + G-CSF ± on-demand PLX | €195.78 | €19.58 ^b^ | | €157.41 | €234.15 | €195.78 | €19.58 | ^9, 10^ |  |
| 110 | uc_INHS_funded_Day_Inw_Hosp_FebNeu_for_CTX_plus_G_CSF_w_wo_PLX_MOB | Parameters (L170) | Unit cost INHS-funded day inpatient hospitalization (febrile neutropenia) for patients on CTX 4 g/m^2^ + G-CSF ± on-demand PLX | €422.07 | €42.21 ^b^ | | €339.34 | €504.79 | €422.07 | €42.21 | ^9, 10^ |  |

*APH* apheresis, *FC* flow cytometry, *CG* caregiver, *CI* confidence interval, *CTX* cyclophosphamide, *CV* coefficient of variation, *CVC* central venous catheter, *DH* day-hospital, *FebNeu* febrile neutropenia, *G-CSF* granulocyte colony-stimulating factor, *INHS* Italian National Health Service, *Inw Hosp* inward hospitalization, *LL* lower limit, *MOB* mobilization, *PLX* plerixafor, *pt* patient, *SE* standard error, *uc* unit cost, *UL* upper limit, *w* with, *wo* without.

^a^A normal distribution was fitted to the unit cost of healthcare resources different from drugs and non-healthcare resources ^4, 5^.

^b^If the analytical calculation of SE was unfeasible, a CV of 10% of the sample mean was imposed ^4-6^.

## Supplementary Table S12 Parameters with normal statistical distribution for base case and sensitivity analyses (one-way and probabilistic)

| **Parameter number** | **Parameter**  **name** | **Spreadsheet**  **(point estimate cell)** | **Parameter**  **description** | **Mean** | **SE** | **95% CI** | | **Normal distribution parameters^a^** | | **Source** |  |
| --- | --- | --- | --- | --- | --- | --- | --- | --- | --- | --- | --- |
|  |  |  |  |  |  | **LL** | **UL** | **Mean** | **SE** |  |  |
| **G-CSF alone vs CTX 4 g/m^2^ + G-CSF (± on-demand PLX)** | | | | | | | | | | |  |
| **Healthcare resources: unit costs (€2019)** | | | | | | | | | | | |
| 111 | uc_INHS_funded_Outpatient_FebNeu_for_CTX_plus_G_CSF_w_wo_PLX_MOB | Parameters (L171) | Unit cost INHS-funded episode (febrile neutropenia) outpatient setting for pts on CTX 4 g/m^2^ + G-CSF ± on-demand PLX | €113.95 | €11.40^b^ | €91.62 | €136.28 | €113.95 | €11.40 | ^11^ |  |
| 112 | uc_INHS_funded_Day_Inw_Hosp_apheresis_for_CTX_plus_G_CSF_w_wo_PLX_APH | Parameters (L172) | Unit cost INHS-funded DH access (apheresis) for pts on CTX 4 g/m^2^ + G-CSF ± on-demand PLX | €211.03 | €21.10^b^ | €169.67 | €252.04 | €211.03 | €21.10 | ^9, 10^ |  |
| 113 | uc_INHS_funded_RBC_bag_CTX_plus_G_CSF_w_wo_PLX_MOB | Parameters (L174) | Unit cost INHS-funded RBC transfusion for pts on CTX 4 g/m^2^ + G-CSF ± on-demand PLX | €153.00 | €15.30^b^ | €123.01 | €182.99 | €153.00 | €15.30 | ^9^ |  |
| 114 | uc_INHS_funded_PLT_bag_CTX_plus_G_CSF_w_wo_PLX_MOB | Parameters (L175) | Unit cost INHS-funded PLT transfusion for pts on CTX 4 g/m^2^ + G-CSF ± on-demand PLX | €115.00 | €11.50^b^ | €92. 46 | €137.54 | €115.00 | €11.50 | ^9^ |  |
| 115 | uc_INHS_funded_RBC_transf_CTX_plus_G_CSF_w_wo_PLX_MOB | Parameters (L176) | Unit cost INHS-funded episode hospitalization (RBC transfusion) for pts on CTX 4 g/m^2^ + G-CSF ± on-demand PLX | €1913.20 | €191.32^b^ | €169.67 | €1538.22 | €2288.18 | €191.32 | ^9, 10^ |  |
| 116 | uc_INHS_funded_RBC_transf_CTX_plus_G_CSF_w_wo_PLX_MOB | Parameters (L177) | Unit cost INHS-funded episode hospitalization (PLT transfusion) for pts on CTX 4 g/m^2^ + G-CSF ± on-demand PLX | €1913.20 | €191.32^b^ | €169.67 | €1538.22 | €2288.18 | €191.32 | ^9, 10^ |  |

*APH* apheresis, *CI* confidence interval, *CVC* central venous catheter, *CG* caregiver, *CF* flow cytometry, *CTX* cyclophosphamide, *CV* coefficient of variation, *DH* day-hospital, *G-CSF* granulocyte colony-stimulating factor, *INHS* Italian National Health Service, *Inw Hosp* inward hospitalization, *LL* lower limit, *MOB* mobilization, *NeuFeb* febrile neutropenia, *PLT* platelet, *PLX* plerixafor, *pt* patient, *RBC* red blood cell, *SE* standard error, *transf* transfusion, *uc* unit cost, *UL* upper limit, *w* with, *wo* without.

^a^A normal distribution was fitted to the unit cost of healthcare resources different from drugs and non-healthcare resources ^4, 5^.

^b^If the analytical calculation of SE was unfeasible, a CV of 10% of the sample mean was imposed ^4-6^.

## Supplementary Table S13 Parameters with statistical distribution for base case and sensitivity analyses (one-way and probabilistic)

| **Parameter number** | **Parameter**  **name** | **Spreadsheet**  **(point estimate cell)** | **Parameter**  **description** | **Mean** | **SE** | **95% CI** | | **Normal distribution parameters^a^** | | | **Source** |  |
| --- | --- | --- | --- | --- | --- | --- | --- | --- | --- | --- | --- | --- |
|  |  |  |  |  |  | **LL** | **UL** | **Mean** | **SE** | |  |  |
| **G-CSF alone vs CTX 4 g/m^2^ + G-CSF (± on-demand PLX)** | | | | | | | | | | | |  |
| **Healthcare resources: unit costs (€2019)** | | | | | | | | | | | | |
| 117 | uc_INHS_funded_SC_processing_APH | Parameters (L179) | Unit cost INHS-funded SC processing (apheresis) | €668.00 | €66.80^b^ | €537.07 | €798.93 | €668.00 | | €66.80 | ^9^ | |
| 118 | uc_INHS_funded_SC_freezing_APH | Parameters (L180) | Unit cost INHS-funded SC cryopreservation (apheresis) | €510.00 | €51.00^b^ | €410.04 | €609.96 | €510.00 | | €51.00 | ^9^ | |
| 119 | uc_INHS_funded_SC_thawing_APH | Parameters (L181) | Unit cost INHS-funded stem cells thawing (apheresis) | €148.00 | €14.80^b^ | €118.99 | €177.01 | €148.00 | | €14.80 | ^9^ | |
| **Non health care resources - Unit costs (€2019)** | | | | | | | | | | | | |
| 120 | uc_Pkg_per_minute | Parameters (L184) | Unit cost per min of hospital parking | €0.05 | €0.01^b^ | €0.04 | €0.06 | €0.05 | | €0.01 | ^12^ | |
| 121 | uc_Car_per_km | Parameters (L185) | Unit cost per km of car | €0.42 | €0.04^b^ | €0.34 | €0.51 | €0.42 | | €0.04 | ^13^ | |
| 122 | uc_minute_Patient_time | Parameters (L186) | Unit cost per min pt’s time | €0.45 | €0.04^b^ | €0.37 | €0.54 | €0.45 | | €0.04 | ^14-17^ | |
| 123 | uc_minute_Caregiver_time | Parameters (L187) | Unit cost per min caregiver’s time | €0.27 | €0.03^b^ | €0.22 | €0.32 | €0.27 | | €0.03 | ^14-17^ | |

*APH* apheresis, *CI* confidence interval, *CTX* cyclophosphamide, *CV* coefficient of variation, *G-CSF* granulocyte colony-stimulating factor, *INHS* Italian National Health Service, *LL* lower limit, *Pkg* parking, *PLX* plerixafor, *SC* stem cell, *SE* standard error, *uc* unit cost, *UL* upper limit, *w* with, *wo* without.

^a^A normal distribution was fitted to the unit cost of healthcare resources different from drugs and non-healthcare resources ^4, 5^.

^b^If the analytical calculation of SE was unfeasible, a CV of 10% of the sample mean was imposed ^4-6^.

## Supplementary Fig. S1 Model outline for (A) G-CSF with on-demand plerixafor and (B) G-CSF without on-demand plerixafor. *G-CSF* granulocyte colony-stimulating factor, *PLT* platelet, *PLX* plerixafor, *RBC* red blood cell.

**A)**

**Pathway probabilities (A)**

**
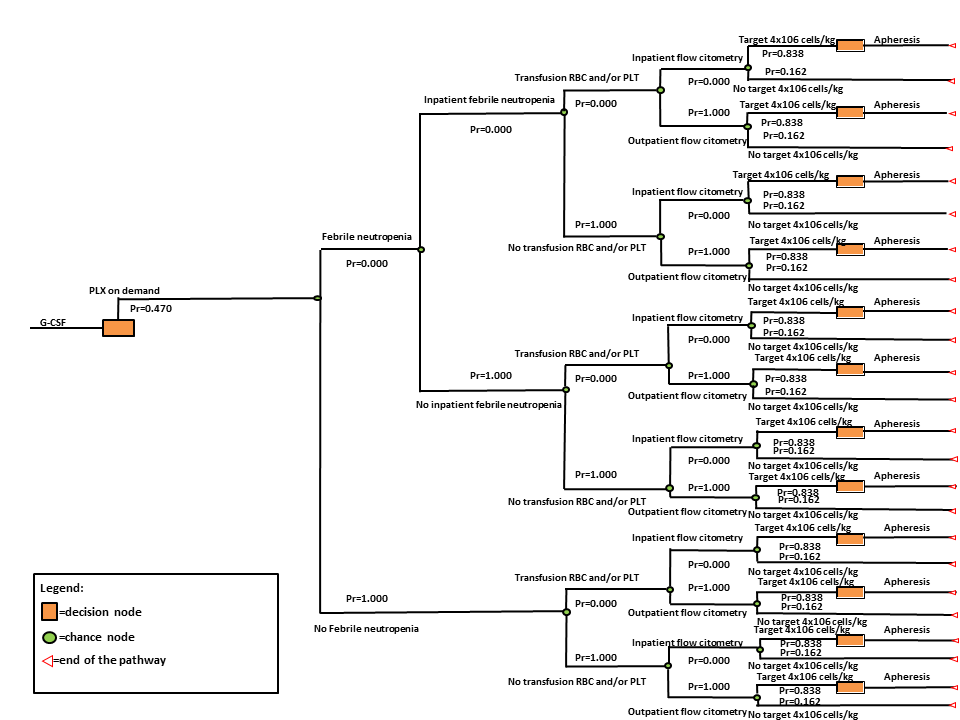
**

**0.39386**

**0.07614**

**0.00000**

**0.00000**

**0.00000**

**0.00000**

**0.00000**

**0.00000**

**0.00000**

**0.00000**

**0.00000**

**0.00000**

**0.00000**

**0.00000**

**0.00000**

**0.00000**

**0.00000**

**0.00000**

**0.00000**

**0.00000**

**0.00000**

**0.00000**

**0.00000**

**0.00000**

**B)**

**Pathway probabilities (B)**

**
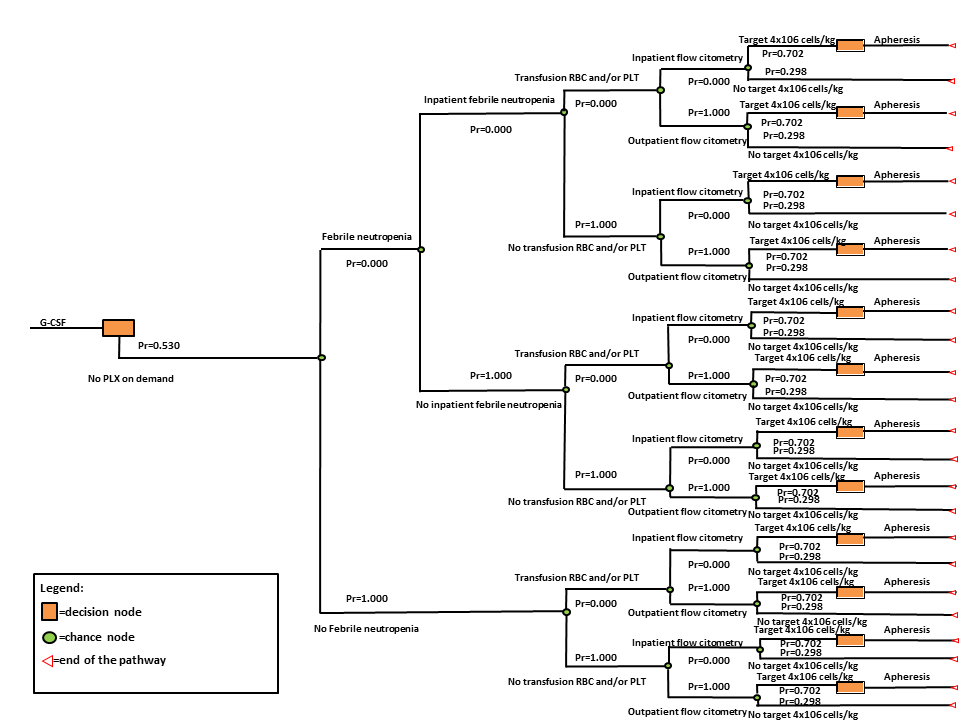
**

**0.00000**

**0.00000**

**0.00000**

**0.00000**

**0.00000**

**0.00000**

**0.00000**

**0.00000**

**0.00000**

**0.00000**

**0.15794**

**0.37206**

**0.00000**

**0.00000**

**0.00000**

**0.00000**

**0.00000**

**0.00000**

**0.00000**

**0.00000**

**0.00000**

**0.00000**

**0.00000**

**0.00000**

Sum pathway probabilities (A)=0.47000; sum pathway probabilities (B)= 0.53000; sum pathway probabilities (A)+(B)=1.00000.

## Supplementary Fig. S2 Model outline for (A) cyclophosphamide 4 g/m^2^ + G-CSF with on-demand plerixafor and (B) cyclophosphamide 4 g/m^2^ + G-CSF without on-demand plerixafor. *CTX* cyclophosphamide, *G-CSF* granulocyte colony-stimulating factor, *PLT* platelet, *PLX* plerixafor, *RBC* red blood cell.

**A)**

**Pathway probability (A)**

**
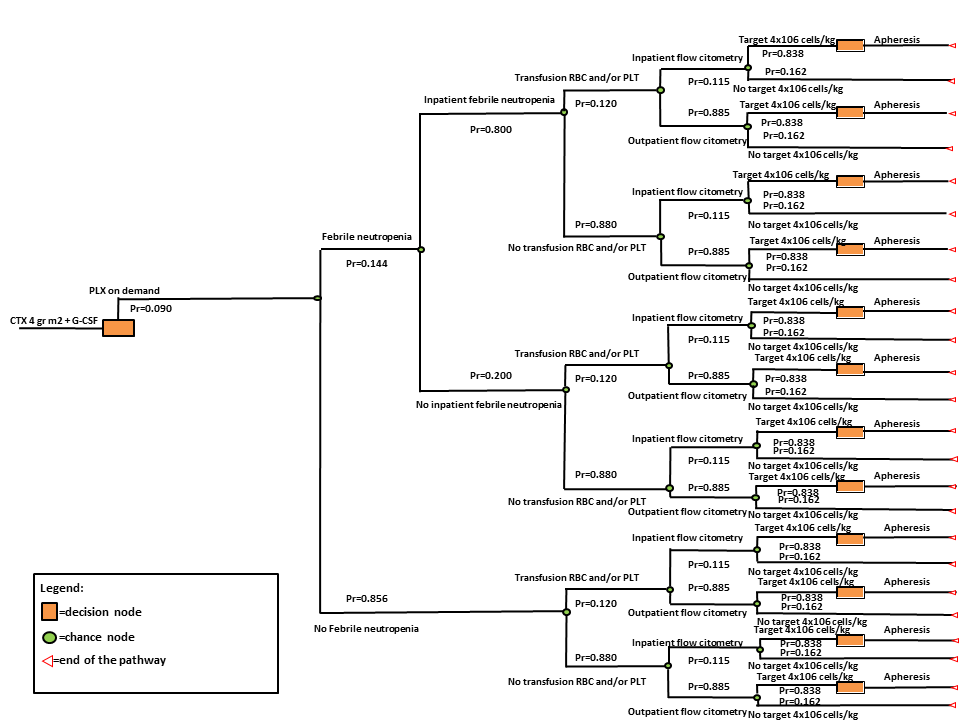
**

**0.00972**

**0.00092**

**0.00018**

**0.00088**

**0.00001**

**0.05028**

**0.00126**

**0.00653**

**0.00133**

**0.00686**

**0.00017**

**0.00089**

**0.00033**

**0.00169**

**0.00004**

**0.00022**

**0.00004**

**0.00023**

**0.00003**

**0.00131**

**0.00677**

**0.00017**

**0.00002**

**0.00012**

**(B)**

**Pathway probabilities (B)**

**0.00012**

**
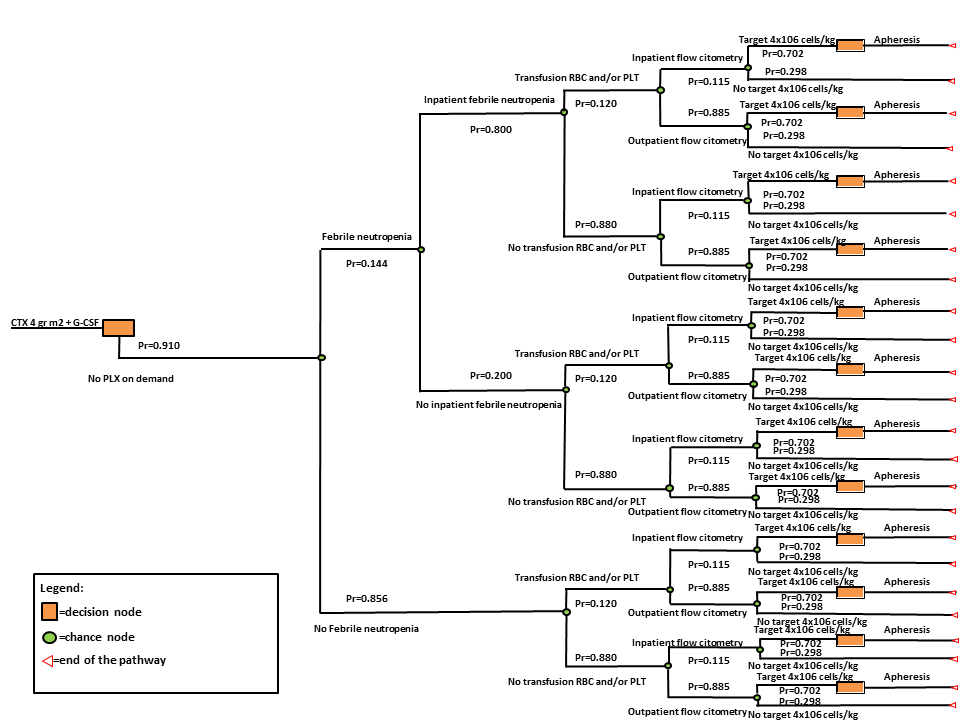
**

**0.18078.02433**

**0.42587.02433**

**0.02349.02433**

**0.05534.02433**

**0.02465.02433**

**0.05807.02433**

**0.00320.02433**

**0.00755.02433**

**0.00608.02433**

**0.01433.02433**

**0.00079.02433**

**0.00186.02433**

**0.00083.02433**

**0.00195.02433**

**0.00011.02433**

**0.00025.02433**

**0.024330.02433**

**0.05731**

**0.00316**

**0.00745**

**0.00332**

**0.00782**

**0.00043**

Sum pathway probabilities (A)=0.19483; sum pathway probabilities (B)= 0.80517; sum pathway probabilities (A)+(B)=1.00000.

## Section B: Cost-effectiveness analysis and sensitivity analysis

**Essential glossary**

**Definition 1. Incremental cost-effectiveness ratio (ICER):** is the most important indicator of cost-effectiveness analysis ^18, 19^, representing the ratio of the difference in cost (incremental cost [ΔC]) between two (or more) healthcare programmes to the difference in effectiveness (incremental effectiveness [ΔE]) between the same options ^18, 19^.

In general, the ICER provides the healthcare decision-makers with the cost for obtaining an incremental unit of effectiveness with the healthcare program with better effect on patient’s health state that is also more costly than the comparator(s) ^18, 19^.

More formally:

$$ICER=\frac{\Delta C}{\Delta E}$$

where:

*ΔC = (CostA – CostB)*

*ΔE = (EffectivenessA –Effectiveness B)*

In order to be cost-effective, the ICER of a given healthcare program should be lower than a given threshold value (λ)

$$ICER<\lambda$$

which is equivalent to:

$$\frac{\Delta C}{\Delta E}<\lambda$$

**Definition 2. Net monetary benefit (NMB):** an algebraic manipulation of the incremental cost-effectiveness ratio (ICER) ^4, 5^.

The NMB is simply a different way of connecting the items included in ICER calculation:

(*NMB_A_ – NMB_B_*) = [(*λ* * *Effectiveness_A_*) – *Cost_A_*] – [(*λ* * *Effectiveness_B_*) – Cost_B_]

Hence, the incremental NMB (ΔNMB) is simply a different representation of the ICER:

*ΔNMB* = [*λ* * (*Effectiveness_A_* – *Effectiveness_B_*)] – (*Cost_A_* – *Cost_B_*)

which is equivalent to:

*ΔNMB* = [(*λ* * *ΔE*) – *ΔC*]

The reversed sign of ICER inequality indicates the condition for the healthcare program under investigation to be cost-effective:

$$\Delta NMB>0$$

**Definition 3. Cost-effectiveness acceptability curve** **(CEAC):** a graphical method that summarizes the uncertainty concerning the baseline estimate of the ICER.

A set of threshold values (eg, from €0 to €100 000) that represent the healthcare policy makers’ willingness to pay for an ΔE are reported on the x-axis, and the probability for the healthcare program under investigation to be cost-effective (as usual bounded between 0–1) is reported on the y-axis.

For each threshold value, healthcare decision-makers can read the probability for the healthcare program under investigation to be cost-effective by looking across from the y-axis to the CEAC ^4, 5, 20^.

**Definition 4. Cost-effectiveness acceptability frontier (CEAF):** an elaboration of cost-effectiveness acceptability curve, the CEAF is a graphical method that represents the probability that the healthcare program with the highest NMB is also cost-effective ^4, 20^.

# References

1. Milone G, Martino M, Leotta S, Spadaro A, Zammit V, Cupri A *et al.* Cost-effectiveness of on-demand plerixafor added to chemotherapy and granulocyte-colony stimulating factor for peripheral blood stem cell mobilization in multiple myeloma. *Leuk Lymphoma* 2018; **59**(1)**:** 42-48. e-pub ahead of print 2017/06/03; doi: 10.1080/10428194.2017.1324161

2. Torrinomedica S.r.l. Ricerca Farmaci [database on the Internet]. Rome: Torrinomedica S.r.l. (in Italian). In, 2019.

3. Sanofi Genzyme. Final report advisory board MM autologous stem cells mobilization PE model: analysis and validation of the most appropriate alternative approaches in Italy. Unpublished report (in Italian). In, 2018.

4. Briggs A, Schulper M, Claxton K. *Decision modelling for health economic evaluation*, Oxford University Press: Oxford, UK, 2006.

5. Briggs AH. Handling uncertainty in economic evaluation and presenting the results. In: Drummond M, McGuire A (eds). *Economic evaluation in health care: merging theory with practice*. Oxford University Press: Oxford, UK, 2001, pp 172-214.

6. Pagano M, Gauvreau K. *Principles of biostatistics*, 2nd edn Duxbury Press: Pacific Grove, USA, 2000.

7. Ministero della Salute. Ministry of Health. Decree 8 February 2013. Criteria for the composition and proper functioning of ethical committees (13A03474). Gazzetta Ufficiale della Repubblica Italiana, Serie Generale, n. 96, 24 April 2013 (in Italian). In.

8. Regione Toscana. Ente per i Serizi Tecnico-Amministrativi di Area Vasta (ESTAV) Centro. Scheda prodotto Catetere venoso centrale ad inserimento periferico con rivestimento in argento [Tuscany Region. Technical and accounting services board for central vast area (ESTAV). Product form on silver-coated peripheral venous catheter]. Firenze: ESTAV Centro, 23 April 2012. In.

9. Standing Conference on the Relations between the State, the Regions and the Autonomous Provinces of Trento and Bolzano. National agreement on tariffs for inter-regional mobility of patients. Version in force for 2014-2015 and 2016. Rome, 2 February 2017 (in Italian). In.

10. Ministero della Salute. Ministry of Health. Annual report on hospitalizations. Data obtained from hospital discharge forms 2017. Rome, January 2019 (in Italian). In.

11. Lazzaro C, Barone C, Caprioni F, Cascinu S, Falcone A, Maiello E *et al.* An Italian cost-effectiveness analysis of paclitaxel albumin (nab-paclitaxel) + gemcitabine vs gemcitabine alone for metastatic pancreatic cancer patients: the APICE study. *Expert Rev Pharmacoecon Outcomes Res* 2018; **18**(4)**:** 435-446. e-pub ahead of print 2018/04/12; doi: 10.1080/14737167.2018.1464394

12. Comune di Milano. Municipality of Milan. Authorized parking for non-residents’ car (in Italian). In, 2019.

13. Agenzia delle Entrate. Italian Revenue Agency. National tariffs per kilometer for cars and motorbikes set by the Auto club of Italy - Art. 3, paragraph 1, of the legislative decree 2 September 1997, n. 314. Gazzetta Ufficiale, Serie Generale, n. 295, 20 December 2018, ordinary supplement n. 57: 9 (in Italian). In.

14. Agenzia delle Entrate. Italian Revenue Agency. The taxpayer yearbook 2013. Rome: Agenzia delle Entrate, October 2013: 51 (in Italian). In.

15. Banca d’Italia. Bank of Italy. Survey on Household Income and Wealth 2016: Table 13 (in Italian). In.

16. Istituto Nazionale per la Previdenza Sociale. National Institute for Social Security. Channel Information. Minima daily wages (in Italian). In.

17. Sistema Statistico Nazionale - Istituto Nazionale di Statistica. National Institute for Statistics. Consumer prices index for the whole nation (reference year 2015=100) - monthly data (in Italian). In.

18. Drummond MF, Schulper MJ, Claxton K, Stoddart GL, Torrance GW. *Methods for the economic evaluation of health care programmes*, 4th edn Oxford University Press: Oxford, UK, 2015.

19. Neumann PJ, Ganiats TG, Russell LB, Sanders GD, Siegel JE (eds). *Cost-effectiveness in health and medicine*. Oxford University Press: New York, USA, 2016.

20. Fenwick E, Claxton K, Sculpher M. Representing uncertainty: the role of cost-effectiveness acceptability curves. *Health Econ* 2001; **10**(8)**:** 779-787. e-pub ahead of print 2001/12/18; doi: 10.1002/hec.635
